# Supplementary material for: Intra-crystalline mesoporous zeolite encapsulation-derived thermally robust metal nanocatalyst in deep oxidation of light alkanes
Source: Nat Commun. 2022 Jan 13;13:295. doi: 10.1038/s41467-021-27828-x (PMC8758710; doi:10.1038/s41467-021-27828-x)
Supplement: Supplementary file 1 — Supplementary Information [file 41467_2021_27828_MOESM1_ESM.pdf]

Supplementary Information

Intra-crystalline mesoporous zeolite encapsulation-derived thermally robust metal nanocatalyst in deep oxidation of light alkanes

Honggen Peng,[1‡✉] Tao Dong,[1‡] Shenyou Yang,[1] Hao Chen,[2, 3] Zhenzhen Yang,[2, 3] Wenming Liu,[1] Chi He,[4✉] Peng Wu,[5] Jinshu Tian,[6] Yue Peng,[7] Xuefeng Chu,[7] Daishe Wu,[1] Taicheng An,[8✉] Yong Wang,[9] Sheng Dai[2, 3✉]

[1] Key Laboratory of Poyang Lake Environment and Resource Utilization, Ministry of Education, School of Resources Environmental and Chemical Engineering, College of Chemistry, Nanchang University, 999 Xuefu Road, Nanchang, Jiangxi 330031, China.

[2] Chemical Sciences Division, Oak Ridge National Laboratory, Oak Ridge, TN 37830, United States.

[3] Department of Chemistry, University of Tennessee, Knoxville, TN 37996, United States.

[4] State Key Laboratory of Multiphase Flow in Power Engineering, School of Energy and Power Engineering, Xi'an Jiaotong University, Xi'an 710049, Shaanxi, China.

[5] Shanghai Key Laboratory of Green Chemistry and Chemical Processes, Department of Chemistry and Molecular Engineering, East China Normal University, North Zhongshan Road 3663, Shanghai 200062, China.

[6] Technology and College of Chemical Engineering, Zhejiang University of Technology, Hangzhou, 310014, China.

[7] State Key Joint Laboratory of Environment Simulation and Pollution Control, National Engineering Laboratory for Multi Flue Gas Pollution Control Technology and Equipment, School of Environment, Tsinghua University, Beijing 100084, China.

[8] Guangdong Key Laboratory of Environmental Catalysis and Health Risk Control, School of Environmental Science and Engineering, Institute of Environmental Health and Pollution Control, Guangdong University of Technology, Guangzhou 510006, China.

[9] The Gene and Linda Voiland School of Chemical Engineering and Bioengineering, Washington State University, Pullman, WA 99164, United States.

List of Contents

Supplementary Methods **1**
Catalysts preparation 1
Catalyst characterization 1
Investigation on growth mechanism of Pd@IM-S-1 2
In situ DRIFTS measuring processes 3
Density functional theory calculations 3
Activity test 3
Reaction kinetics measurement 4
Thermal stability test, water resistance and recycling test 4
Supplementary Tables **6**
Supplementary Table 1. Physicochemical properties and Pd content of related catalysts are measured by N_2 sorption isotherms and ICP. 6
Supplementary Table 2. EXAFS fitting parameters at the Pd K-edge for samples (S_0^2 = 0.70)..7
Supplementary Table 3. Catalytic activities of Pd@IM-S-1 and related catalysts. 8
Supplementary Table 4. Comparison with literatures about the performances of Pd based catalysts for CH_4 or C_3H_8 oxidation. 9
Supplementary Table 5. The parameters of Weisz-Prater criterion (C_{WP}) for internal diffusion of Pd@IM-S-1 and related catalysts for CH_4 oxidation process. 10
Supplementary Table 6. The parameters of Weisz-Prater criterion (C_{WP}) for internal diffusion of Pd@IM-S-1 and related catalysts for C_3H_8 oxidation process. 10
Supplementary Table 7. The parameters of Mears' criterion (C_M) for external diffusion of of Pd@IM-S-1 and related catalysts for CH_4 oxidation process. 10
Supplementary Table 8. The parameters of Mears' criterion (C_M) for external diffusion of of Pd@IM-S-1 and related catalysts for C_3H_8 oxidation process. 11
Supplementary Table 9. Kinetic catalytic performances of Pd@IM-S-1 and related catalysts. [a] 12
Supplementary Table 10. Summary of the vibrational modes and positions of surface species in the oxy-carbon surface species region (1300-1800 cm^{-1}) of propane oxidation over Pd@IM-S-1. 13
Supplementary Figures **14**
Supplementary Fig. 1 Comparison with the previous synthetic pathways of zeolite-confined metal nanoparticles (NPs) catalysts. 14
Supplementary Fig. 2 TEM images of Pd@SiO_2. 15
Supplementary Fig. 3 XRD patterns of Pd@IM-S-1 and related catalysts. 16
Supplementary Fig. 4 TEM images of Pd@IM-S-1. 17
Supplementary Fig. 5 SEM images of Pd@IM-S-1. 18
Supplementary Fig. 6 HRTEM and selected area electron diffraction (SAED) of Pd@IM-S-1. 19
Supplementary Fig. 7 HAADF-STEM, elemental EDS Mapping images and line scan of Pd@IM-S-1. 20
Supplementary Fig. 8 TEM images of and Pd/S-1. 21
Supplementary Fig. 9 FIB specimens preparation process of Pd@IM-S-1. 22
Supplementary Fig. 10 FIB-AC-TEM, FIB-AC-BF-TEM, FIB-AC-EDS-mapping, larger Pd NPs size

distribution of Pd@IM-S-1. 23
Supplementary Fig. 11 N_2 adsorption/desorption isotherms of Pd@IM-S-1 and related catalysts. 24
Supplementary Fig. 12 XRD patterns of SiO_2 and IM-S-1. 25
Supplementary Fig. 13 TEM of SiO_2 and IM-S-1. 26
Supplementary Fig. 14 XRD patterns of 1%-Pt@SiO_2 and 1%-Pt@IM-S-1. 27
Supplementary Fig. 15 TEM of 1%-Pt@SiO_2 and 1%-Pt-@IM-S-1 28
Supplementary Fig. 16 Tomogram-section TEM images and Pt NPs size distribution of 1%-Pt@IM-S-1. 29
Supplementary Fig. 17 XRD patterns of 1%-Rh@SiO_2 and 1%-Rh@IM-S-1. 30
Supplementary Fig. 18 TEM of 1%-Rh@SiO_2 and 1%-Rh@IM-S-1. 31
Supplementary Fig. 19 Tomogram-section TEM and HAADF-STEM images, and Rh NPs size distribution of 1%-Rh@IM-S-1. 32
Supplementary Fig. 20 XRD patterns of 1%-Ru@SiO_2 and 1%-Ru@IM-S-1. 33
Supplementary Fig. 21 TEM of 1%-Ru@SiO_2 and 1%-Ru@IM-S-1. 34
Supplementary Fig. 22 Tomogram-section TEM images and Ru NPs size distribution of 1%-Ru@IM-S-1. 35
Supplementary Fig. 23 Raman spectra of unreduced samples PdO@IM-S-1, PdO/S-1, PdO@SiO_2 and reduced samples Pd@IM-S-1, Pd/S-1, Pd@SiO_2. 36
Supplementary Fig. 24 The optimized structure of Pd (111), PdO (101) and Pd-PdO interface. 37
Supplementary Fig. 25 Oxygen dissociation process on Pd (111) slab....................................38
Supplementary Fig. 26 Oxygen vacancy formation energy (E_v) with different structure models. 39
Supplementary Fig. 27 The activation energy (E_a) of the methane first C-H cleavage over the Pd (111) model. 40
Supplementary Fig. 28 The activation energy (E_a) of the methane first C-H cleavage over the PdO (101) model. 41
Supplementary Fig. 29 The activation energy (E_a) of the methane first C-H cleavage over the Pd-PdO interface model. 42
Supplementary Fig. 30 The possible reaction pathways of methane first C-H cleavage over these different structure models. 43
Supplementary Fig. 31 Deep oxidation performances of methane and propane over PdO@IM-S-1 and Pd@IM-S-1. 44
Supplementary Fig. 32 XRD patterns and Raman spectra of unreduced sample PdO@IM-S-1, unreacted sample Pd@IM-S-1-fresh, and reacted sample Pd@IM-S-1-used. 45
Supplementary Fig. 33 XPS of Pd3d for Pd@IM-S-1-fresh and Pd@IM-S-1-used. 46
Supplementary Fig. 34 Tomogram-section TEM images and Pd NPs size distribution of Pd@IM-S-1-800. 47
Supplementary Fig. 35 Regeneration test of Pd/S-1 in the oxidation of propane. 48
Supplementary Fig. 36 In situ DRIFTS spectra collected during propane deep oxidation over Pd@IM-S-1 at 325 °C. 49
Supplementary Fig. 37 In situ DRIFTS spectra collected during propane temperature programed desorption (50 – 400 ^oC) in a N_2 + O_2 atmosphere over Pd@IM-S-1...................50

Supplementary Fig. 38 In situ DRIFTS spectra collected from the C_3H_8 + O_2 reaction over Pd@IM-S-1 between 50 ^oC and 400 ^oC......51
Supplementary Fig. 39 Possible reaction mechanism for the oxidation of propane on Pd@IM-S-1 catalyst. 52
Supplementary References......53

Supplementary Methods

Catalysts preparation

Synthesis of Pd@SiO_2. Pd@SiO_2 was synthesized by using a reversed-phase microemulsion method according to our previous report[1,2] with some modifications. Briefly, a calculated amount of 4.32 mL $Pd(NH_3)_4(NO_3)_2$ solution was rapidly added into 960 mL of cyclohexane solution containing NP-5 (polyethylene glycol mono-4-nonylphenyl ether, 40.32 g). After stirring at 25 ^oC for 15 h, an aqueous ammonia solution (28 wt.%, 4.32 mL) was then added quickly. After stirring for 2 h, tetraethyl orthosilicate (TEOS, 5 mL) was added into the above mixture solution. Next, the solution was stirred for 48 h at 25 ^oC. The initial sample was collected by centrifugation at 9000 rpm. After drying in a vacuum oven at 40 °C overnight, and then calcining at 550 °C for 4 h in air, the final sample of Pd@SiO_2 was obtained.

Synthesis of SiO_2, Pt@SiO_2, Rh@SiO_2 and Ru@SiO_2. The synthesis of SiO_2, Pt@SiO_2, Rh@SiO_2 and Ru@SiO_2 were similar to the Pd@SiO_2, and respectively, only the precursor solution were replaced by H_2O, $Pt(NH_3)_4(NO_3)_2$ solution, $RhCl_3$ solution and $RuCl_3$ solution.

Synthesis of Pd@IM-S-1. The Pd@IM-S-1 was synthesized by in situ conversion of the SiO_2 shell in Pd@SiO_2 to silicalite-1 through a continuous heating in situ dry-gel conversion method according to previously reported literature[3,4] with some modifications. For typical synthesis of Pd@IM-S-1, 0.4 g of Pd@SiO_2 and 0.5332 g of tetrapropylammonium hydroxide (TPAOH, 25 wt.%) was mixing and then subject to grinding at room temperature for 15 min. The solid was dried under infrared light for 15 min to partly remove the water, and then the solid powder was transferred into a Teflon-lined autoclave with limited amount of water. The material was thermally treated in a conventional oven at 80 ^oC for 2 days and then at 120 ^oC for 1 day under static conditions. Finally, the material was calcined at 550 ^oC for 4 h to obtain the Pd@IM-S-1 sample.

Synthesis of IM-S-1, Pt@IM-S-1, Rh@IM-S-1 and Ru@IM-S-1. The IM-S-1, Pt@IM-S-1, Rh@S-1 and Ru@IM-S-1 were synthesized by in situ dry-gel conversion method was similar to the preparation of Pd@IM-S-1.

Synthesis of S-1. Typically, 20 mL TEOS was firstly mixed with 16 mL TPAOH (25 wt.%) and then 44 mL deionized water was added into the above mixture solution at room temperature with fast stirring until hydrolyzed completely, followed transferred into a Teflon-lined autoclave and conducted in a conventional oven at 170 ^oC for 1 day. The initial sample was collected by centrifugation at 9000 rpm and washed with deionized water for several times. After drying in a conventional oven at 80 °C overnight and calcining at 550 °C for 6 h in air, the final sample of S-1 was obtained.

Synthesis of Pd/S-1. For a comparison study, Pd/S-1 was prepared by Pd nanoparticles supported on the outer surface of S-1 (The S-1 was not calcined.). As a typical run for the synthesis of Pd/S-1 (The content of Pd is consistent with Pd@IM-S-1 sample.) were prepared via wet impregnation method. After impregnation, the samples were dried in a conventional oven at 80 °C overnight and calcined at 550 °C for 4 h in air, the final sample of Pd/S-1 was obtained.

Catalyst characterization

X-ray diffraction (XRD) patterns were recorded on a Bruker AXS D8Focus diffractometer that operated at 40 kV and 30 mA with a Cu target and Kα-ray irradiation (λ = 1.54178 Å). Scans were collected in the 2θ range from 5 to 65 ° with a step of 2 ° min^{-1} to analyze the phase structure. Raman spectra were collected in the anti-Stokes range of 200-1500 cm^{-1} using an inVia Reflex-Renishaw spectrometer. The Pd and Ce content of catalysts was determined by inductively coupled plasma optical emission spectrometry (ICP-OES) were performed with an Agilent 5100 ICP OES with Dichroic Spectral Combiner (DSC) technology (Agilent Technologies, Mulgrave, Australia). Scanning electron microscope (SEM), transmission electron microscope (TEM), scanning TEM (STEM), high-resolution TEM (HRTEM), line scan and elemental mapping images were obtained using a JEOL 3000F TEM working at 300 kV equipped with an energy dispersive spectroscopy (EDS) detector. The Tomogram-section TEM images were obtained using a Leica EM UC7 working at FEI Talos F200X 200kV. Samples for TEM investigation were prepared by dispersing samples in ethanol and immersing them in an ultrasonic bath for 30 min, and then dropping a few drops of the resulting suspensions onto a copper grid coated with a layer of amorphous carbon. Nitrogen adsorption-desorption analysis was carried out at 77 K on Micromeritics ASAP2020 instrument. The

specific surface areas of the samples were calculated using Brunauer-Emmett-Teller (BET) method. The total pore volume of each catalyst was accumulated at a relative pressure of P/P_0 = 0.99. X-ray photoelectron spectrum (XPS) was performed on PHI-5000C ESCA system using a single Mg-K-X-ray source operated at 250 W and 14 kV voltage. The spectra were obtained at ambient temperature with an ultra-high vacuum. The binding energies were calibrated by the standard C1s peak of graphite at 284.8 eV.

To confirm Pd@IM-S-1 has possess abundant intra-mesopores and Pd NPs are confined within the mesopores of the zeolite shell. The focused ion beam (FIB) technique was performed to preparing specimens for aberration-corrected TEM characterization. The FIB specimens were obtained using the FEI Helios Nanolab 600i from ZKKF (BEIJING) Science & Technology Co., Ltd. and working at 500 V–30 kV. Detailed steps are as follows:

(1) **Sample pretreatment:** Attached non-conductive powder sample Pd@IM-S-1 to a carbon film.

(2) **Selecting area and depositing Pt protective layer:** In the electron beam imaging mode of the double beam electron microscope, select the area of interest, and use the electron beam to deposit a layer of C or Pt with a thickness of about 0.5-1 microns, and then use the ion beam to deposit a layer of Pt with a thickness of 2-3 microns to protect the surface structure of the sample.

(3) **Proposal and transfer:** Gradually dig out V-shaped grooves on both sides and cut off the root of the sample, extend the manipulator and weld the top of the sample, then cut the supporting parts on both sides of the sheet, take out the sample and transfer it to the support and weld it firmly. Finally, the sample was cut and separated from the tip of the manipulator.

(4) **Thinning:** The FIB specimens were obtained through the ion beam to thinning the thin sample layer. The actual thinning parameters, including the acceleration voltage required to change the thickness and the tilt angle of the sample during the thinning process, mainly depend on the material system being studied. For general materials, the following parameters: use 30 KV to process the sample to ~1000 nm, then reduce the voltage to 16 KV, continue to thin to ~500 nm, and then reduce the voltage to 8 KV, continue to thin to ~200nm, and finally use 5 KV to thin to ~100 nm. If the sample needs to be characterized with high resolution, it is also necessary to reduce the voltage to 2 KV and gradually reduce the thickness of the sample to ~50 nm. After thinning to the target thickness, the sample is cleaned with a low voltage of 1 KV to remove the amorphous layer introduced by the ion beam.

The simple flowchart was presented in **Figure S8.** Hereafter, the aberration-corrected TEM (AC-TEM) was applied to characterize FIB specimens via the FEI titan themis working at 300 kV equipped with an energy dispersive spectroscopy (EDS) detector.

The X-ray absorption spectra were collected on the beamline BL01C1 in National Synchrotron Radiation Research Center (NSRRC), with electron energy of 1.5 GeV and a beam current between 100 and 200 mA, and were provided technical support by Ceshigo Research Service "www.ceshigo.com". The radiation was monochromatized by a Si (111) double-crystal monochromator. The XAFS data were recorded under fluorescence mode with standard Lytle ion chambers for the Pd K-edge. Typically, the energy was calibrated according to the absorption edge of a pure Pd foil as appropriate. The Athena software was used to extract and fit the XANES and EXAFS data. The passive electron factors, S_0^2, were determined by fitting the experimental Pd foil data and fixing the Pd–Pd coordination number (CN) to be 12, and then fixed for further analysis of the measured samples. In addition, the parameters including CN, bond distance (R), Debye-Waller factor (σ^2), and the inner potential correction (ΔE_0) were describing the electronic properties and local structure environment.

The *in situ* near ambient pressure X-ray photoelectron spectroscopy (*In situ* NAP-XPS) measurements were carried out on a SPECS NAP-XPS system. All spectra were obtained using monochromatized Al Kα irradiation (1,486.6 eV) generated by an Al anode (SPECS XR-50) and an excitation source power fixed at 50 W. XPS measurements at pressures up to ~25 mbar are possible owing to a differential pumping system, which separates the electron analyser (SPECS Phoibos NAP-150) from the reaction area. The aperture of the nozzle is 0.3 mm. Through a pressure reducing valve kept the reaction pressure was at 1 mbar (C_3H_8:O_2 = 1:5).

Investigation on growth mechanism of Pd@IM-S-1

To understand the growth mechanism of Pd@IM-S-1, the crystallinity and morphological evolution with different crystallization time between 80 to 120 ^oC has been monitored. First, the Pd@IM-S-1-80-1d, Pd@IM-S-1-80-2d, Pd@IM-S-1-120-1h, Pd@IM-S-1-120-2h, Pd@IM-S-1-120-4h, Pd@IM-S-1-120-12h and Pd@IM-S-1-120-24h samples are obtained by in situ dry-gel conversion method. These samples are thermally treated in a conventional oven as following 80 ^oC for 1 day→80 ^oC for 2 days→120 ^oC for 1 hour→120 ^oC for 2 hours→120 ^oC for 4 hours→120 ^oC for 12 hours→120 ^oC for 24 hours under static conditions. And then, the XRD and TEM are applied to characterization the crystallinity and morphological evolution of these samples. During

this test, all samples are unreduced.

In situ DRIFTS measuring processes

Propane adsorption and oxidation at 325 °C. *In situ* diffuse reflectance infrared Fourier transform spectroscopy (*In situ* DRIFT) measurements were performed of Pd@IM-S-1 sample on a Bruker Vertex FT-IR spectrometer with a mercury-cadmium-telluride (MCT) detector. In the DRIFT cell with KBr windows connected with a gas flow system, the sample was pretreated at 400 °C in N_2 for 30 min and then cooled to 325 °C in N_2. After the background spectra were recorded at the temperature, N_2 gas was replaced by the gas of 0.2% C_3H_8-99.8% N_2 (30 mL min^{-1}), and in situ DRIFT spectra of the samples were taken at different times. After 30 min, the gas of 0.2% C_3H_8-99.8% N_2 was replaced by the gas of 10% O_2-90% N_2 (30 mL min^{-1}) flow for 30 min.

Propane temperature-programmed desorption in N_2 + O_2 atmosphere. The TPD experiments were performed on the same Bruker Vertex FT-IR spectrometer. The sample powders of Pd@IM-S-1 were first purged in situ in a N_2 stream (30 mL min^{-1}) at 400 °C for 30 min and then cooled to 50 °C in N_2. After the background spectra were recorded at 50 °C, N_2 gas was replaced by the gas of 0.2% C_3H_8-99.8% N_2 was fed at a flow rate of 30 mL min^{-1} for 30 min and pure N_2 was introduced to remove any physical adsorption molecules. The spectra were recorded from 50 to 400 °C in 10% O_2-90% N_2 atmosphere at a heating rate of 10 °C min^{-1}.

Propane temperature-programmed oxidation. The IR experiment of the reaction pathway was also recorded on the Bruker Vertex FT-IR spectrometer. The sample powders of Pd@IM-S-1 were purged in situ in a N_2 stream (30 mL min^{-1}) at 400 °C for 30 min and then cooled to 50 °C in N_2. After the background spectra were recorded at 50 °C, N_2 gas was replaced by the gas of 0.2% C_3H_8-10% O_2-89.8% N_2 was fed at a flow rate of 30 mL min^{-1}. The spectra were recorded from 50 to 400 °C at a heating rate of 10 °C min^{-1}.

Density functional theory calculations

The periodic density function theory (DFT) calculations were carried out using the CP2K package.[5,6] The exchange-correlation potential was described using the Perdew-Burke-Ernzerh (PBE) form of the generalized-gradient approximation (GGA).[7] The wave functions were expanded in a molecularly optimized double-Gaussian basis set, with an auxiliary plane wave basis set with a cutoff energy of 500 Rydberg. The scalar relativistic norm-conserving pseudo-potentials were employed to modelled the core electrons with 18, 6, 4 and 1 valence electrons for Pd, O, C and H, respectively.[8] The only Γ-point in reciprocal space mesh was used for integrating the Brillouin zone. Grimme's third generation DFT-D3 approach was used to describe dispersion corrections.[9] The Pd (111) and PdO (101) surfaces were used to model the Pd and PdO substrates, constructed with cell dimensions of 19.0847×19.0811×29.4223 Å and 12.5225×22.3838×25.7640 Å respectively with 15 Å vacuum space to minimize the interaction between slabs. For the Pd-PdO interface model, we firstly built Pd (111) slab, then generated one layer of PdO (111) on the surface of Pd (111) and finally formed the interface structure shown in **Figure S23**. For the O_2 dissociation on Pd (111), we have also conducted corresponding calculations shown in **Figure S24**. The convergence criterion used for geometry optimizations was a maximum force of 0.01 eV Å^{-1}. Spin polarization was considered in all calculations.

Activity test

Methane (CH_4) and propane (C_3H_8) total oxidation. Methane and propane as the typical light alkanes were selected as the model compounds to evaluate the catalytic activity of Pd@IM-S-1 and related catalysts. First, 50 mg of catalyst (40-60 mesh) was put in a quartz tubular fixed-bed reactor and in situ reduction at 400 °C for 2 h under 10% H_2/Ar (30 mL min^{-1}). Then, the temperature was cooled down to 30 °C and purged for 30 min under ultra-high purity N_2. Subsequently, the activity was test from 200-400 °C at a rate of temperature change of 2 °C min^{-1} under 1% CH_4-21% O_2-N_2 or 0.2% C_3H_8-10% O_2-N_2. The total gas flow rate was 30 mL min^{-1} and the weight hourly space velocity (WHSV) was fixed at 36 000 mL $g_{cat.}^{-1}h^{-1}$. Finally, the reactants and products were monitored through a Fourier transform infrared (FTIR) spectrometer (ANTARIS IGS-Analyzer) with a 2 m gas cell. The CH_4 and C_3H_8 conversion were calculated according to the following equation:

$$X_{HCs} = \frac{[HCs]_{in} - [HCs]_{out}}{[HCs]_{in}} \times 100\% \quad (1)$$

where the $[HCs]_{in}$ and $[HCs]_{out}$ express the inlet and outlet HCs (CH_4 or C_3H_8) concentrations in the feed stream, respectively.

Reaction kinetics measurement

The kinetics data for CH_4 and C_3H_8 combustion of Pd@IM-S-1, Pd/S-1 and Pd@SiO_2 samples were tested in a fixed-bed reactor (101.325 kPa) according to previous reported literature.[10] Typically, the feed gas were consisted of 1% CH_4-21% O_2-N_2 and 0.2% C_3H_8-10% O_2-N_2 with a WHSV of 180 000 mL $g_{cat.}^{-1}h^{-1}$ and 20 mg of catalyst mixed with 80 mg of inert quartz sand (40-60 mesh) was used for each testing while the internal and external diffusion have been eliminated. The methane and propane conversions were controlled within 15%.

The r_{CH_4} and $r_{C_3H_8}$ (mol $(g_{cat}\cdot s)^{-1}$) expressed the reaction rate were calculated by the equation of $r_{CH_4} = X_{CH_4} \cdot V_{CH_4}/g_{cat}$ and $r_{C_3H_8} = X_{C_3H_8} \cdot V_{C_3H_8}/g_{cat}$, where X_{CH_4} and $X_{C_3H_8}$ are the conversion, V_{CH_4} and $V_{C_3H_8}$ are the gas flow rate (mol s^{-1}) of CH_4 and C_3H_8, g_{cat} is the weight of the catalyst.

The total turnover frequency (TOF) (s^{-1}) of CH_4 and C_3H_8 were calculated by the equation of $TOF_{total} = X_{CH_4} \cdot V_{CH_4} \cdot N_A/N_{total}$ and $TOF_{total} = X_{C_3H_8} \cdot V_{C_3H_8} \cdot N_A/N_{total}$, where N_A is Avogadro constant and N_{total} is total atom numbers of Pd, which can be calculated by the equation of $N_{total} = (m_{pd}/M_{pd}) \cdot N_A$, where m_{pd} is the mass and M_{pd} is relative atomic mass of Pd.

Absence of mass transport during kinetic measurements.

The absence of mass transport resistances was checked by Weisz-Prater criterion (C_{WP}) for internal diffusion and Mears' criterion (C_M) for external diffusion.

$$C_{wp} = \frac{r_{obs}\rho_c R_p^2}{D_{eff}C_s} < 1 \quad (2)$$

$$C_M = \frac{r_{obs}\rho_b R_p n}{k_c C_{Ab}} < 0.15 \quad (3)$$

Weisz-Prater Criterion for Internal Diffusion

If $C_{wp} = \frac{r_{obs}\rho_c R_p^2}{D_{eff}C_s} < 1$, then internal mass transfer effects can be neglected.

Where r_{obs} = observed reaction rate, mol/$kg_{cat}\cdot s$

R_p = catalyst particle radius, m

ρ_c = density of catalyst, kg/m^3

D_{eff} = effective diffusivity, m^2/s

C_s = gas concentration of A at the external surface of the catalyst, mol/m^3

The results of internal mass transfer are presented in **Table S6** and **Table S7**.

Mears Criterion for External Diffusion

If $C_M = \frac{r_{obs}\rho_b R_p n}{k_c C_{Ab}} < 0.15$, then external mass transfer effects can be neglected.

Where r_{obs}= observed reaction rate, mol/$kg_{cat}\cdot s$

n= reaction order

R_p= catalyst particle radius, m

ρ_c = density of catalyst, kg/m^3

ρ_b = bulk density of catalyst bed, kg/m^3

= (1-Φ) ρ_c (Φ=porosity)

$\approx \rho_c \approx \rho_{cat}$

C_s = gas concentration of A at the external surface of the catalyst, mol/m^3.

C_{Ab} = bulk gas concentration of A, mol/m^3.

$\approx C_s$

k_c = external mass transfer coefficient, m/s

The results of external mass transfer are presented in **Table S8** and **Table S9**.

Thermal stability test, water resistance and recycling test

Thermal stability test. The Pd@IM-S-1 and Pd/S-1 catalysts were subjected to thermal stability testing to evaluate the sintering resistance performance of the catalysts. Firstly, these two catalysts were calcined at 800 °C for 8 h in argon before testing (noted as Pd@IM-S-1-800 and Pd/S-1-800). These two catalysts were then

activated in-situ at 400 ^{o}C for 2 h under gas composition of 10% H_2 and 90% Ar (30 mL min^{-1}) and the activity was evaluated under gas composition of 0.2% C_3H_8-10% O_2-N_2.

Water resistance test. Water resistance test of Pd@IM-S-1 and Pd/S-1 for propane oxidation was used to evaluate the tolerance to water of catalysts (noted as Pd@IM-S-1-H_2O and Pd/S-1-H_2O). These two catalysts were activated in-situ at 400 ^{o}C for 2 h under gas composition of 10% H_2 and 90% Ar (30 mL min^{-1}). Then, the activity was evaluated under gas composition of 5% H_2O + 0.2% C_3H_8-10% O_2-N_2.

Recycling test. Pd@IM-S-1 was subjected to five reaction cycles under the same conditions, and activated in-situ at 400 ^{o}C for 2 h under gas composition of 10% H_2 and 90% Ar (30 mL min^{-1}) and the activity was evaluated under gas composition of 0.2% C_3H_8-10% O_2-N_2. For each cycle, the temperature was increased from 200 to 400 ºC and then cooled to 200 ^{o}C. Furthermore, after the fresh sample (designated as Pd@IM-S-1-fresh) was subject to five cycling tests (designated as Pd@IM-S-1-5th-run), it was then subjected to a simple regeneration step (reduced at 400 ^{o}C for 2 h in 10% H_2 and 90% Ar, designated as Pd@IM-S-1-restore). The activity of Pd@IM-S-1-5th-run was evaluated under gas composition of 0.2% C_3H_8-10% O_2-N_2.

Supplementary Tables

Supplementary Table 1. Physicochemical properties and Pd content of related catalysts are measured by N_2 sorption isotherms and ICP.

Samples	S_{BET} (m^2 g^{-1})[a]	S_{ext} (m^2 g^{-1})[b]	V_{micro} (cm^3 g^{-1})[c]	D_{micro} (nm)[c]	V_{meso} (cm^3 g^{-1})[d]	D_{meso} (nm)[d]	Pd content (wt.%)[e]
Pd@IM-S-1	408	76	0.158	0.68	0.136	2.5 (20)[d]	1.83
Pd@IM-S-1-800	385	66	0.129	0.58	0.179	2.4 (4.0)	-
Pd/S-1	424	-	0.141	0.59	-	-	1.84
Pd@SiO_2	93	-	0.014	1.4	0.407	36.6	1.73
S-1	452	-	0.167	0.74	-	-	-

[a] Calculated by BET method.

[b] S_{ext} (external surface area) calculated using the t-plot method.

[c] Determined by HK method.

[d] Determined by BJH method

[e] Obtained from ICP.

- Not provided.

Supplementary Table 2. EXAFS fitting parameters at the Pd K-edge for samples (S_0^2 = 0.70).

Samples	Shell	CN[a]	R(Å)[b]	σ^2(Å^2)[c]	ΔE_0(eV)[d]	R factor
Pd foil	Pd-Pd	12	2.74±0.01	0.0051	-0.7±0.5	0.0048
Pd@IM-S-1	Pd-O	0.9±0.3	1.99±0.02	0.0025	-0.1±0.8	0.0085
	Pd-Pd	7.7±0.3	2.74±0.01	0.0052		
	Pd-O-Pd	2.0±1.4	3.41±0.03			
PdO	Pd-O	4.5±0.2	2.03±0.01	0.0012	-0.9±1.1	0.0058
	Pd-Pd	8.3±0.5	3.07±0.01	0.0068		
	Pd-O-Pd	9.4±0.7	3.45±0.01			

[a] CN: coordination numbers; [b]R: bond distance; [c]σ^2: Debye-Waller factors; [d]ΔE_0: the inner potential correction. R factor: goodness of fit. S_0^2 was set to 0.70, according to the experimental EXAFS fit of Pd foil reference by fixing CN as the known crystallographic value.

Supplementary Table 3. Catalytic activities of Pd@IM-S-1 and related catalysts.

Samples	Methane conversion temperatures		Propane conversion temperatures	
	T_{50} (^oC)	T_{90} (^oC)	T_{50} (^oC)	T_{90} (^oC)
Pd@IM-S-1	261	318	260	284
Pd/S-1	280	325	270	297
Pd@SiO_2	306	525	298	334

Supplementary Table 4. Comparison with literatures about the performances of Pd based catalysts for CH_4 or C_3H_8 oxidation.

Catalysts	Pd wt.%	Reaction conditions			T_{90} (°C)	References
		CH_4 (%)	C_3H_8 (%)	WHSV (mL g^{-1} h^{-1})		
**Pd@IM-S-1**	**1.83**	**1**	-	**36000**	**318**	**This work**
		-	**0.2**		**289**	
Pd/Al-Ce(550)	1	0.1	-	50400 h^{-1}	> 400	Appl. Catal. B Environ. 264 (2020) 118475
PdSiCe	0.92	0.5	-	180000 h^{-1}	> 600	Appl. Catal. A Gen. 574 (2019) 79-86
Pd/NA-Al_2O_3	**5**	1	-	15000	> 325	Nat. Commun. 10 (2019) 1611
		-	0.2	30000	~ 300	
Pd/CeO_2	1	-	0.3	240000	401	Appl. Catal. B Environ. 226 (2018) 585-595
Pd/CZ/A	2	-	0.012	40000 h^{-1}	> 300	J. Ind. Eng. Chem. 58 (2018) 246-257
Pd/CeO_2	0.77	1	-	15000	332	Catal. Sci. Technol. 8 (2018) 2567
$Pd_{0.78}Mn_{0.22}$	0.5	0.5	-	20000	400	J. Am. Chem. Soc. 139 (2017) 11989-11997
Pd/Al_2O_3	1.6	-	0.06	40000 h^{-1}	315	Rare Metal Mat. Eng. 46 (2017) 1231-1236
$Co_{3.5}$Pd/3DOM CeO_2	0.4	2.5	-	40000	520	J. Catal. 342 (2016) 17-26
PdO/CeO_2@HZSM-5	0.93	0.5	-	30000	> 535	Nanoscale 8 (2016) 9621-9628
Pd/Al_2O_3	3	-	0.175	300000	~ 375	Catal. Today 201 (2013) 19-24
Pd/ZSM-5	1.5	-	0.2	30000	~ 334	ACS Catal. 3 (2013) 1154-1164
$Pd_x/W_y/TiO_2$	2	-	0.5	45000 h^{-1}	345	J. Catal. 285 (2012) 103-114
Pd@CeO_2/Al_2O_3	1	0.5	-	200000	> 350	Science 337 (2012), 713
Pd/V/Al_2O_3	0.5	-	0.5	45000 h^{-1}	385	Catal. Sci. Technol. 1 (2011) 1367-1375

Supplementary Table 5. The parameters of Weisz-Prater criterion (C_{WP}) for internal diffusion of Pd@IM-S-1 and related catalysts for CH_4 oxidation process.

Samples	ρ_c (kg/m^3)	$C_s \times 10^2$ (mol/m^3)	$R_p \times 10^4$ (m)	$r_{obs} \times 10^4$ (mol/kg$_{cat}$·s)	$D_{eff} \times 10^6$ (m^2/s)	C_{WP}	C_{WP} compare 1
Pd@IM-S-1	542	44.5	1.35	26	8.9	0.006	<1
Pd/S-1	567	44.5	1.35	13	8.9	0.003	<1
Pd@SiO_2	683	44.5	1.35	3.8	8.9	0.001	<1

Supplementary Table 6. The parameters of Weisz-Prater criterion (C_{WP}) for internal diffusion of Pd@IM-S-1 and related catalysts for C_3H_8 oxidation process.

Samples	ρ_c (kg/m^3)	$C_s \times 10^2$ (mol/m^3)	$R_p \times 10^4$ (m)	$r_{obs} \times 10^4$ (mol/kg$_{cat}$·s)	$D_{eff} \times 10^6$ (m^2/s)	C_{WP}	C_{WP} compare 1
Pd@IM-S-1	542	8.9	1.35	3.7	8.9	0.005	<1
Pd/S-1	567	8.9	1.35	1.5	8.9	0.002	<1
Pd@SiO_2	683	8.9	1.35	0.5	8.9	0.001	<1

Because C_{wp} of CH_4 and C_3H_8 over the Pd@IM-S-1 and related catalysts are both below 1, thus the internal mass transfer effects can be neglected.

Supplementary Table 7. The parameters of Mears' criterion (C_M) for external diffusion of of Pd@IM-S-1 and related catalysts for CH_4 oxidation process.

Samples	ρ_b (kg/m^3)	$C_{Ab} \times 10^2$ (mol/m^3)	n	$R_p \times 10^4$ (m)	$r_{obs} \times 10^4$ (mol/kg$_{cat}$·s)	k_c (m^2/s)	C_M	C_M compare 0.15
Pd@IM-S-1	542	44.5	2	1.35	26	0.071	0.012	<0.15
Pd/S-1	567	44.5	2	1.35	13	0.071	0.006	<0.15
Pd@SiO_2	683	44.5	2	1.35	3.8	0.071	0.002	<0.15

Supplementary Table 8. The parameters of Mears' criterion (C_M) for external diffusion of of Pd@IM-S-1 and related catalysts for C_3H_8 oxidation process.

Samples	ρ_b (kg/m^3)	C_{Ab}×10^2 (mol/m^3)	n	R_p×10^4 (m)	r_{obs}×10^4 (mol/kg$_{cat}$·s)	k_c (m^2/s)	C_M	C_M compare 0.15
Pd@IM-S-1	542	8.9	2	1.35	3.7	0.071	0.009	<0.15
Pd/S-1	567	8.9	2	1.35	1.5	0.071	0.004	<0.15
Pd@SiO$_2$	683	8.9	2	1.35	0.5	0.071	0.001	<0.15

Because C_M of CH_4 and C_3H_8 over the Pd@IM-S-1 and related catalysts are both below 0.15, thus the external mass transfer effects can be neglected.

Based on the above results (**Supplementary Table5-8**), internal and external diffusion effects during the kinetic experiment could be neglected.

Supplementary Table 9. Kinetic catalytic performances of Pd@IM-S-1 and related catalysts. [a]

Samples	Ea ($kJ\ mol^{-1}$)		Rate ($mol\ g_{cat.}^{-1}s^{-1}$)		TOF_{total} ($10^3\ s^{-1}$)	
	Methane	Propane	Methane (265 ^oC)	Propane (265 ^oC)	Methane (265 ^oC)	Propane (265 ^oC)
Pd@IM-S-1	114.2	137.2	2.6×10^{-6}	3.7×10^{-7}	14.9	2.14
Pd/S-1	121.9	144.4	1.3×10^{-6}	1.5×10^{-7}	7.4	0.84
Pd@SiO_2	171.9	157.8	3.8×10^{-7}	4.8×10^{-8}	2.4	0.30

[a] Kinetic test conditions: The kinetics data for CH_4 and C_3H_8 oxidation were tested in a fixed-bed reactor at atmospheric pressure. The feed gas were consisted of 1% CH_4-21% O_2-N_2 and 0.2% C_3H_8-10% O_2-N_2 with a WHSV of 180 000 mL $h_{cat.}^{-1}\ g^{-1}$, and 20 mg of catalyst mixed with 80 mg of inert quartz sand (40-60 mesh) was used for each testing while the internal and external diffusion have been eliminated. The methane and propane conversions were controlled within 15%.

Supplementary Table 10. Summary of the vibrational modes and positions of surface species in the oxy-carbon surface species region (1300-1800 cm^{-1}) of propane oxidation over Pd@IM-S-1.

Species	Vibrational mode	Band position (cm^{-1})		Reference
		This work	Literature range	
Acetate	$V_{as}(COO)$	1511,1513,1516,1550	1504-1590	[10-14]
	$V_s(COO)$	1458,1461,1462	1427-1470	[10-14]
Acetone	V(C=O)	1688,1690,1696	1688-1725	[11,15]
Aliphatic ester	V(C=O)	1743,1750,1751	1720-1753	[11,15]
Bicarbonate	$V_{as}(OCO)$	1648,1650	1646-1653	[11,16,17]
Formate	$V_{as}(COO)$	1594,1595	1586-1597	[11,16,18]
Water	δ (HOH)	1648,1650	1636-1650	[11,16,17]

Supplementary Figures

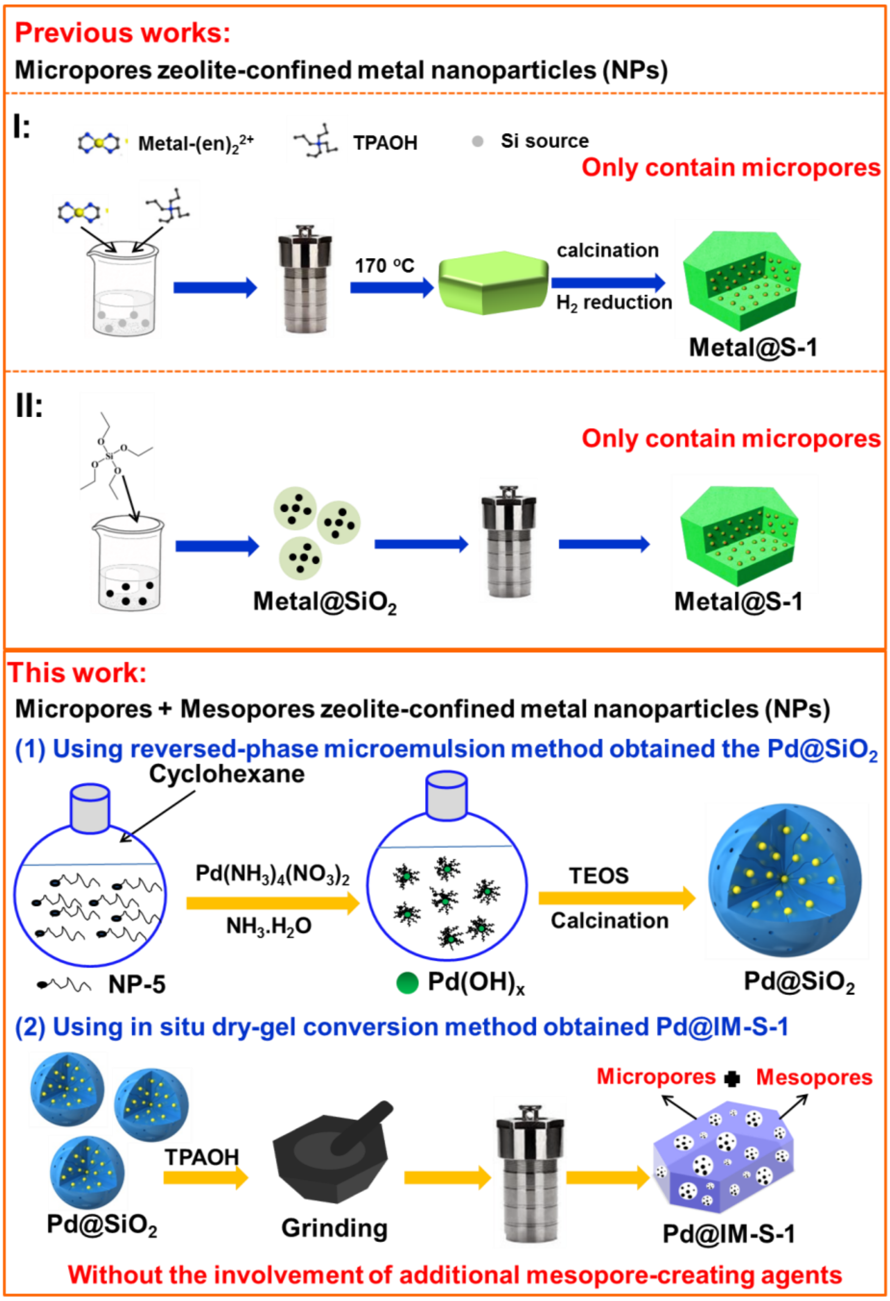

Supplementary Fig. 1 Comparison with the previous synthetic pathways of zeolite-confined metal nanoparticles (NPs) catalysts.

Supplementary Note 1: In this work, we developed a facile in situ mesoporogen-free strategy to design and synthesize palladium (Pd) NPs enveloped within a single-crystalline zeolite (silicalite-1, S-1) with abundant intra-mesopores (termed Pd@IM-S-1). The synthesis steps are as follows: (1) using a reversed-phase microemulsion method obtained the Pd@SiO_2 precursor, (2) the Pd@IM-S-1 sample was prepared by in situ conversion of the amorphous SiO_2 shell of Pd@SiO_2 to silicalite-1 shell through a continuous heating in situ dry-gel conversion method without the involvement of additional mesopore-creating agents. Our work was different from the previous works which are only containing the single microporous channels, and our work contains micropores and the supernumerary intra-mesopores simultaneously.

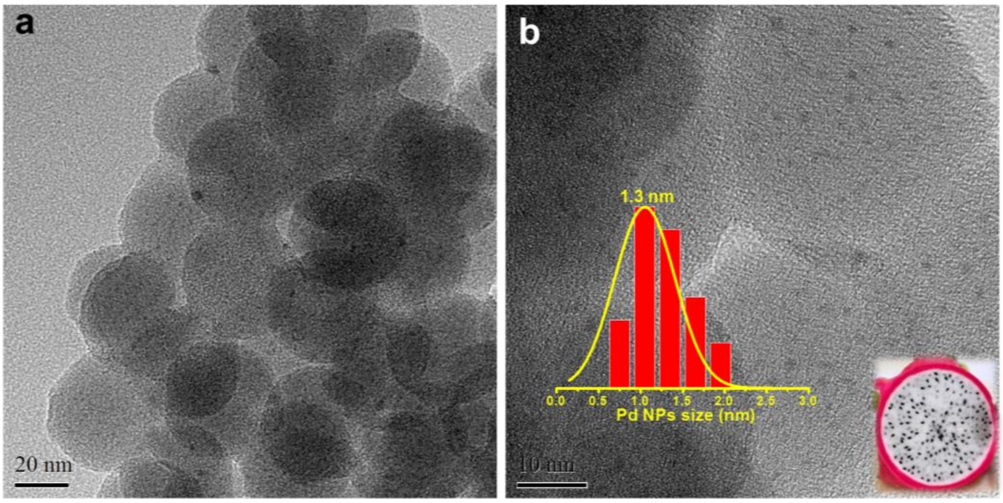

Supplementary Fig. 2 TEM images of Pd@SiO_2 (a, b).

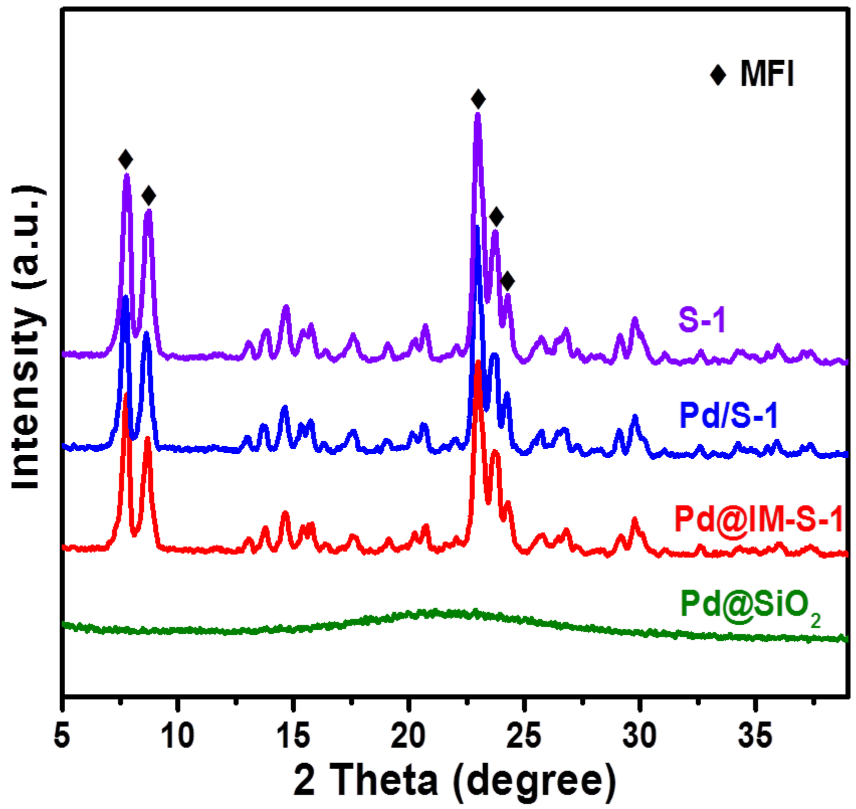

Supplementary Fig. 3 XRD patterns of Pd@IM-S-1 and related catalysts.

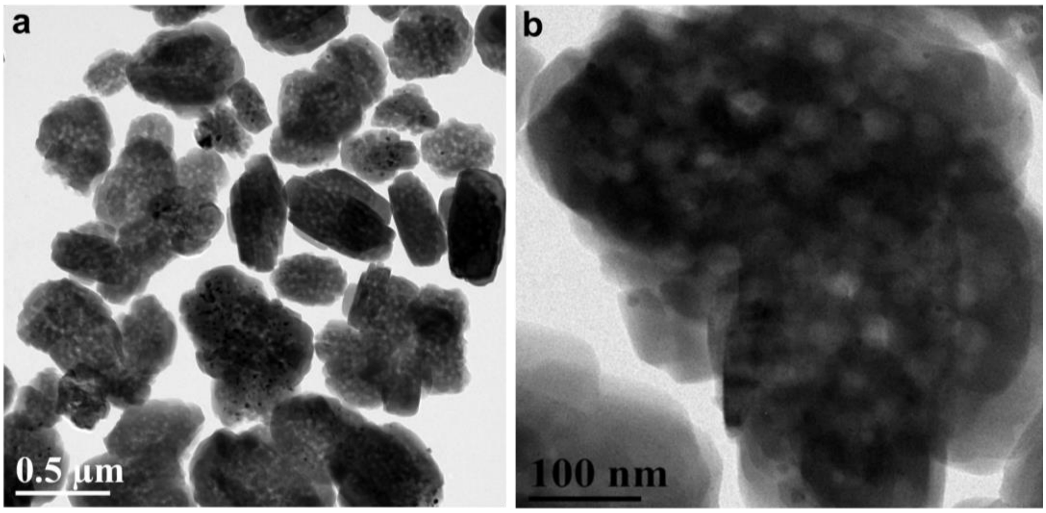

Supplementary Fig. 4 TEM images of Pd@IM-S-1 (a, b).

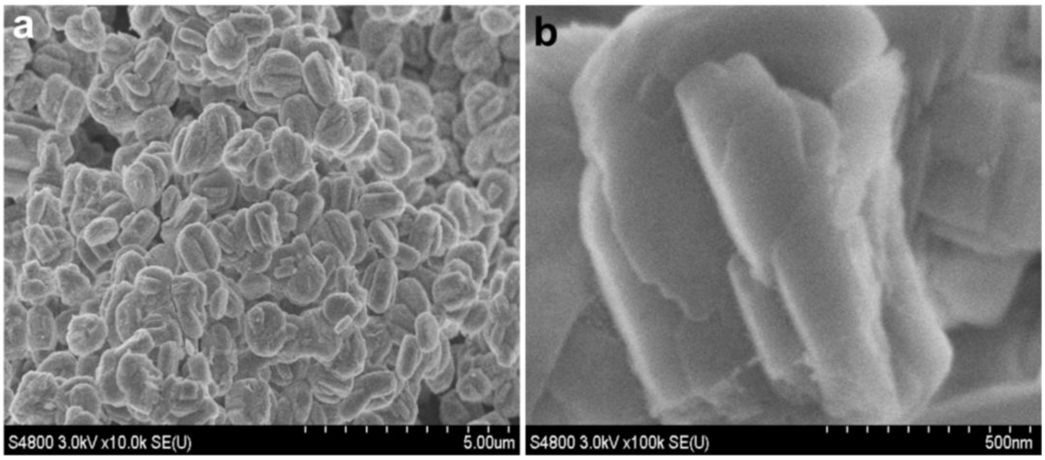

Supplementary Fig. 5 SEM images (a and b) of Pd@IM-S-1.

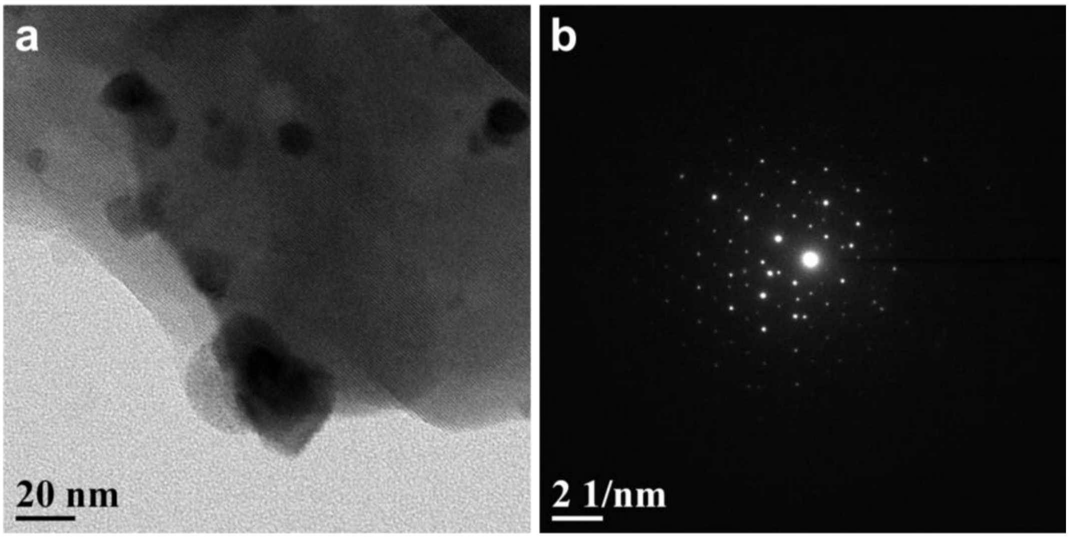

Supplementary Fig. 6 HRTEM (a) and selected area electron diffraction (SAED) (b) of Pd@IM-S-1.

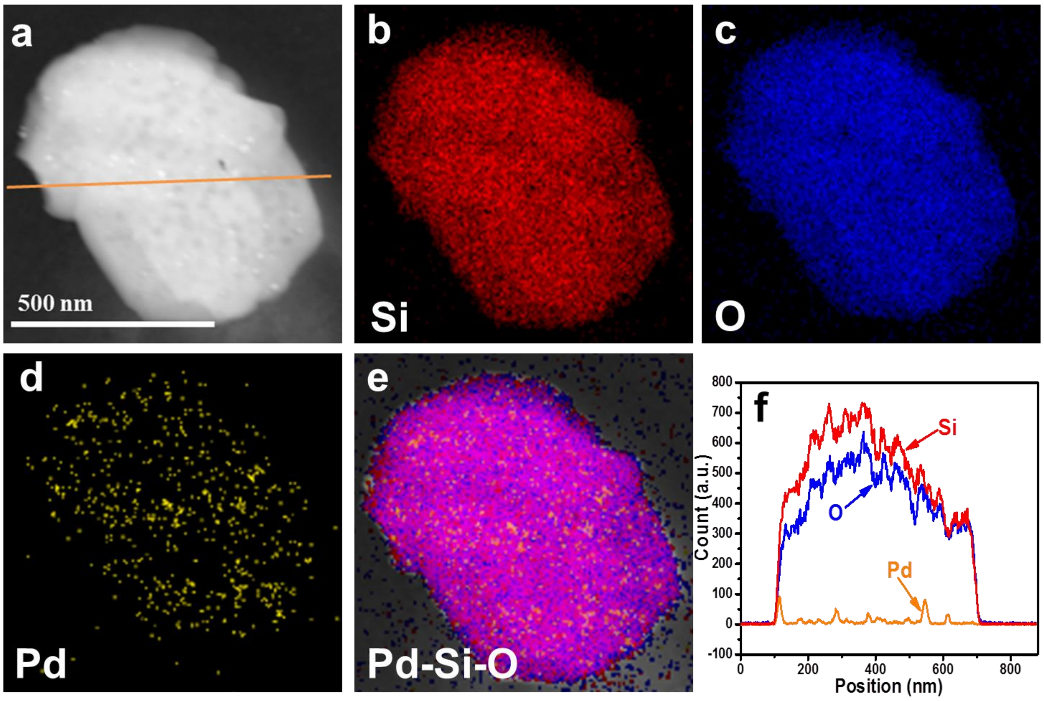

Supplementary Fig. 7 HAADF-STEM (a), elemental EDS Mapping (b-e) images and line scan (f) of Pd@IM-S-1.

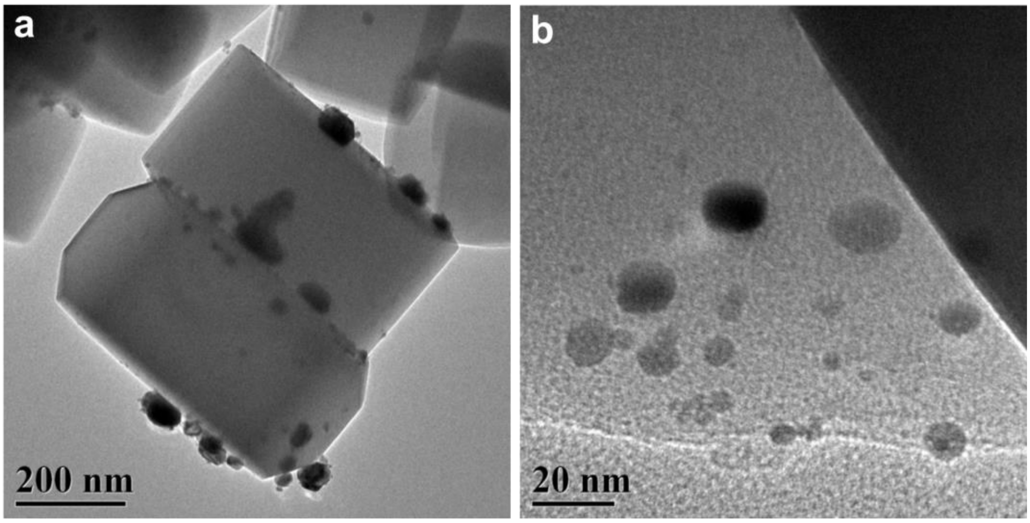

Supplementary Fig. 8 TEM images of and Pd/S-1 (a, b).

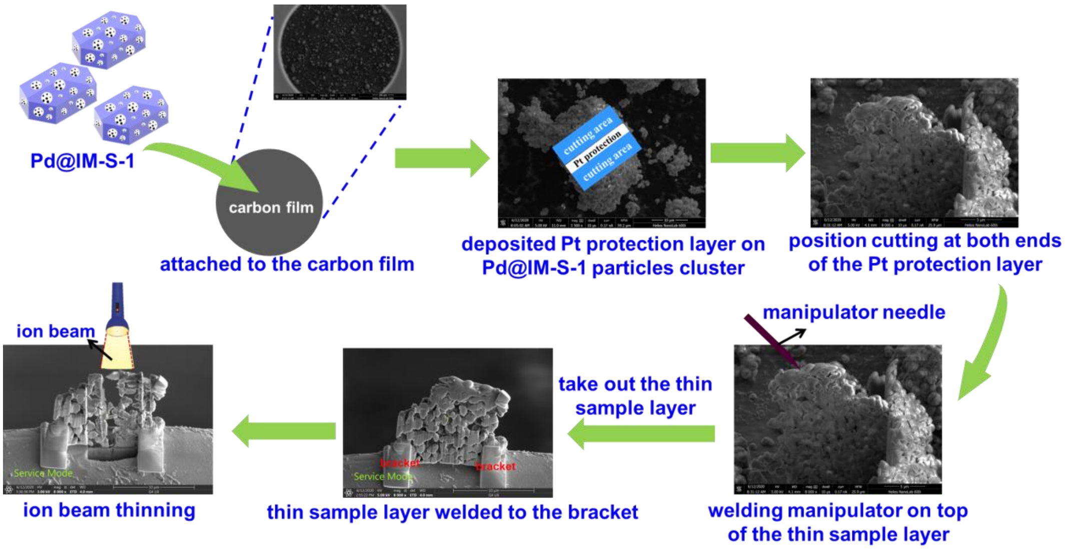

Supplementary Fig. 9 FIB specimens preparation process of Pd@IM-S-1.

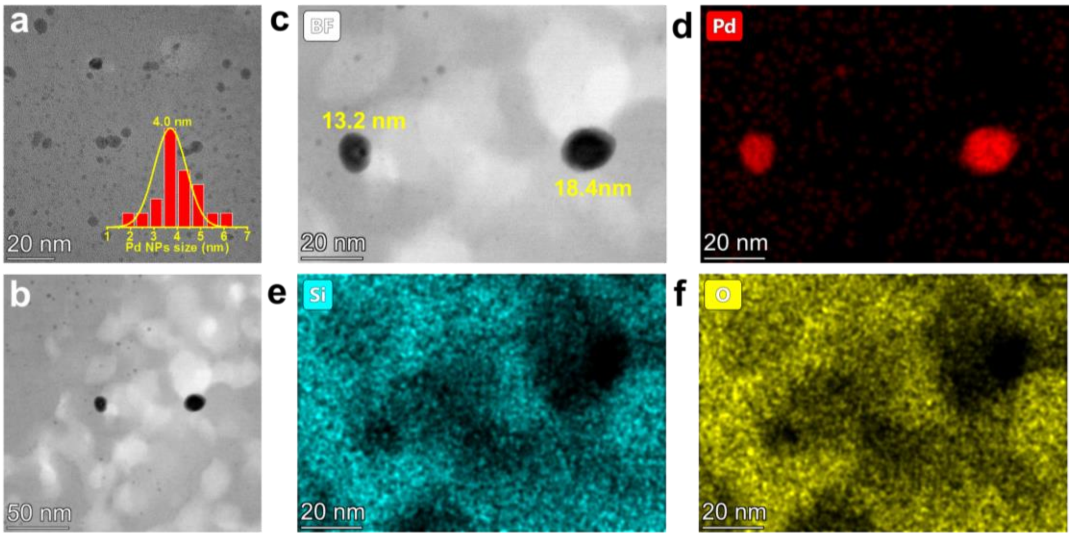

Supplementary Fig. 10 FIB-AC-TEM (a), FIB-AC-BF-TEM (b, c), FIB-AC-EDS-mapping (d-f), larger Pd NPs size distribution (a image internal) of Pd@IM-S-1.

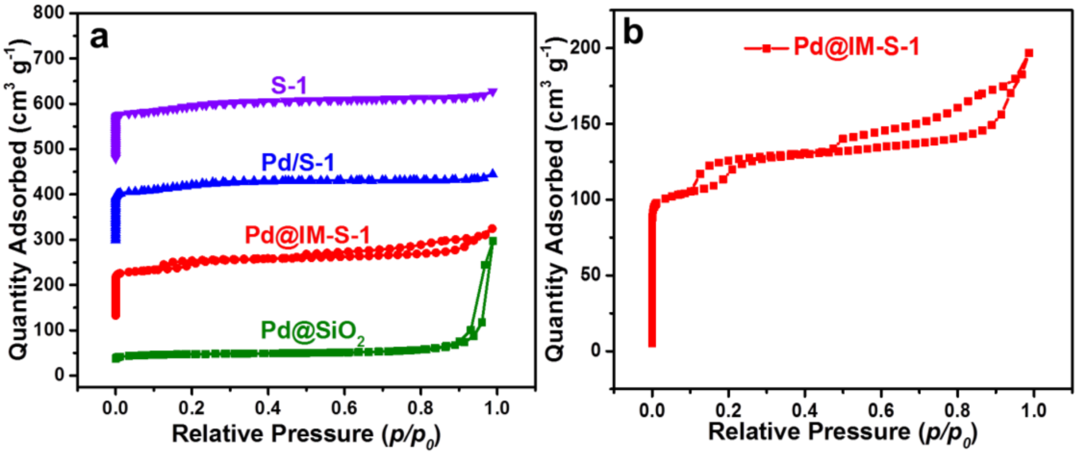

Supplementary Fig. 11 N_2 adsorption/desorption isotherms of Pd@IM-S-1 and related catalysts (a, b).

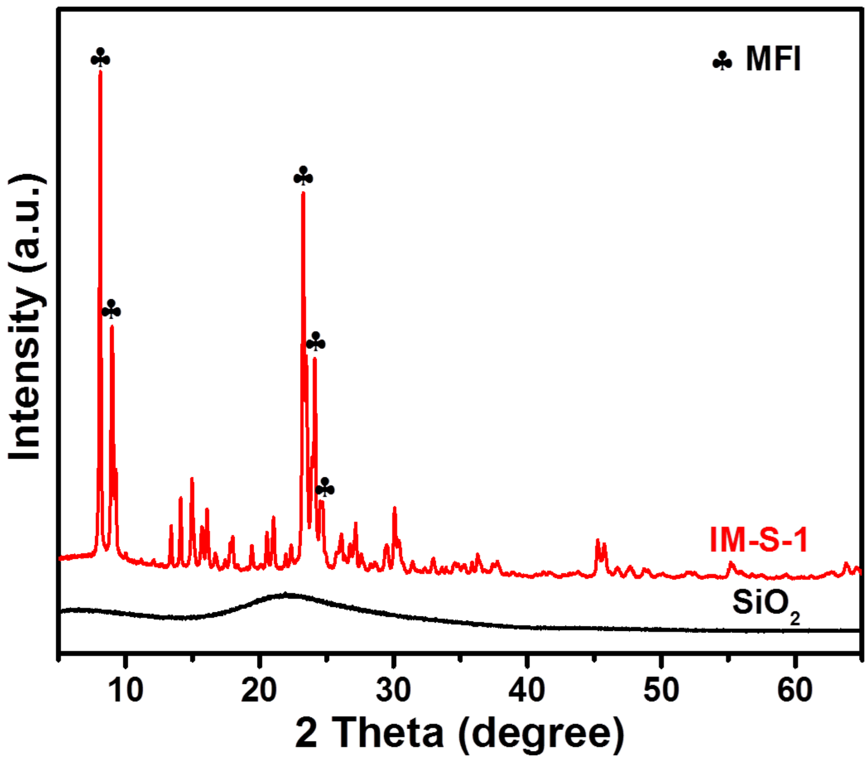

Supplementary Fig. 12 XRD patterns of SiO_2 and IM-S-1.

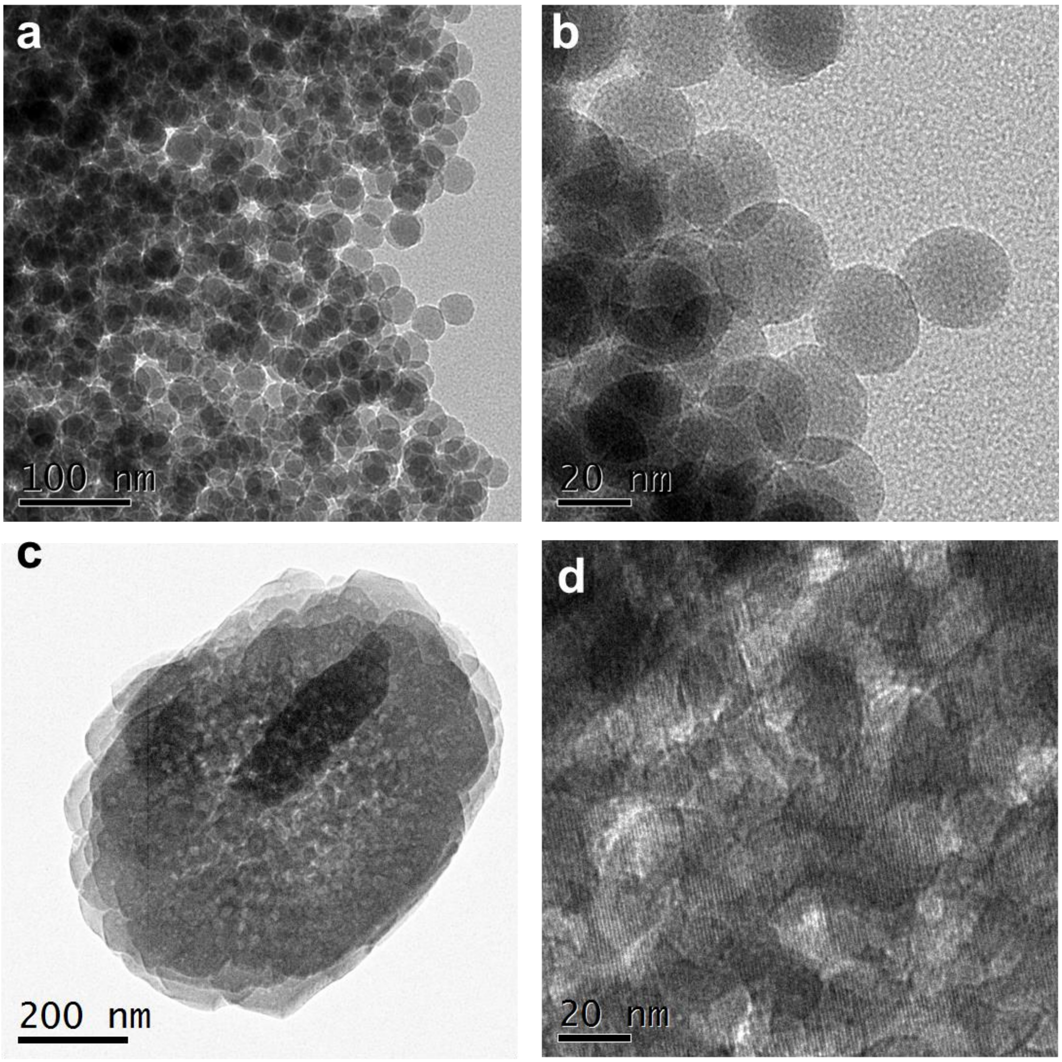

Supplementary Fig. 13 TEM of SiO_2 (a, b) and IM-S-1 (c, d).

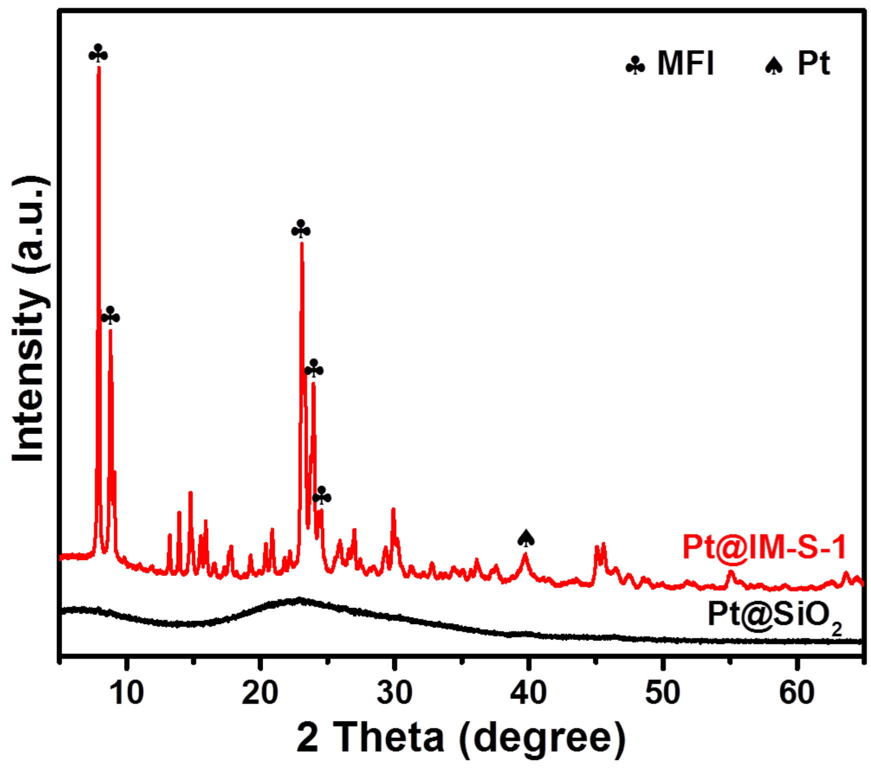

Supplementary Fig. 14 XRD patterns of 1%-Pt@SiO_2 and 1%-Pt@IM-S-1.

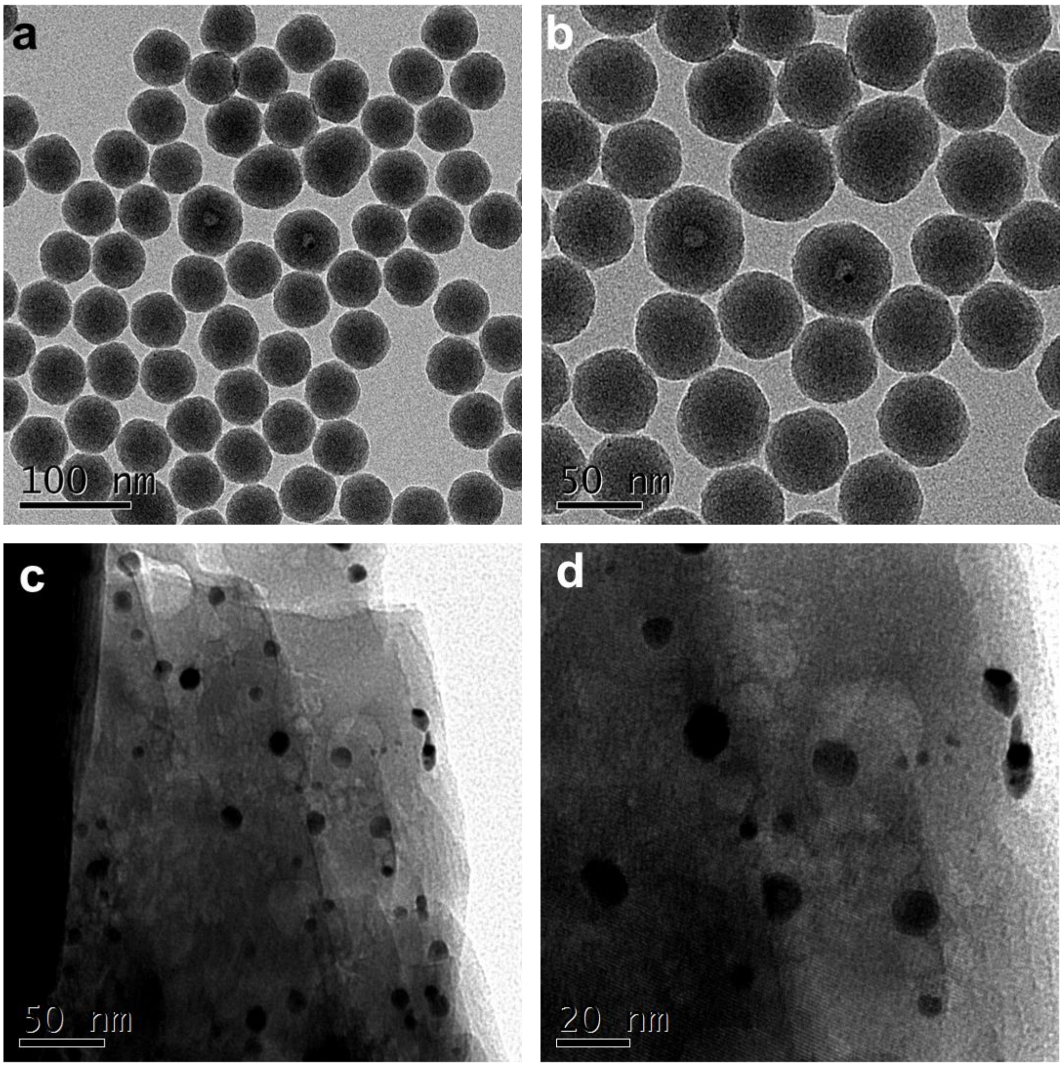

Supplementary Fig. 15 TEM of 1%-Pt@SiO_2 (a, b) and 1%-Pt-@IM-S-1 (c, d).

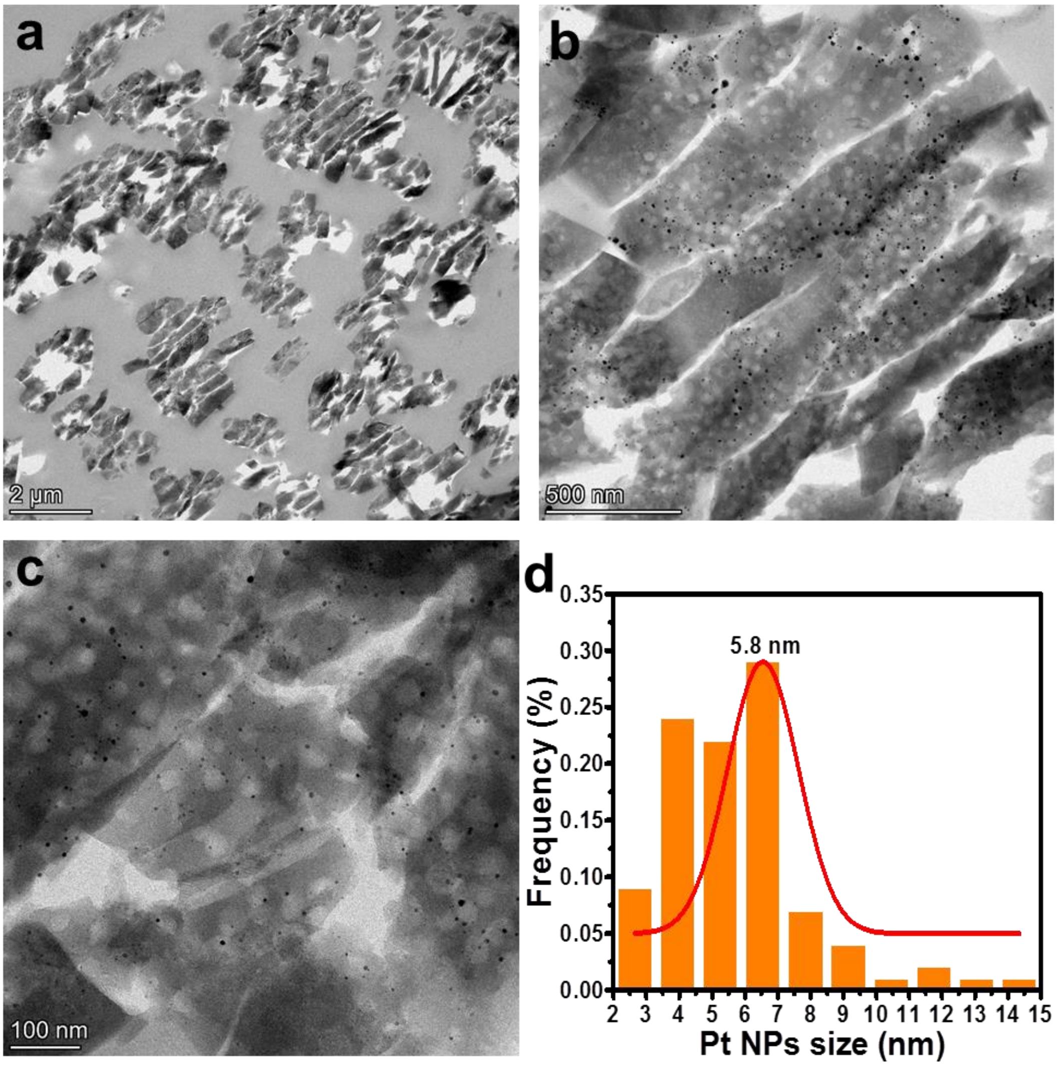

Supplementary Fig. 16 Tomogram-section TEM images (a, b, c) and Pt NPs size distribution (d) of 1%-Pt@IM-S-1.

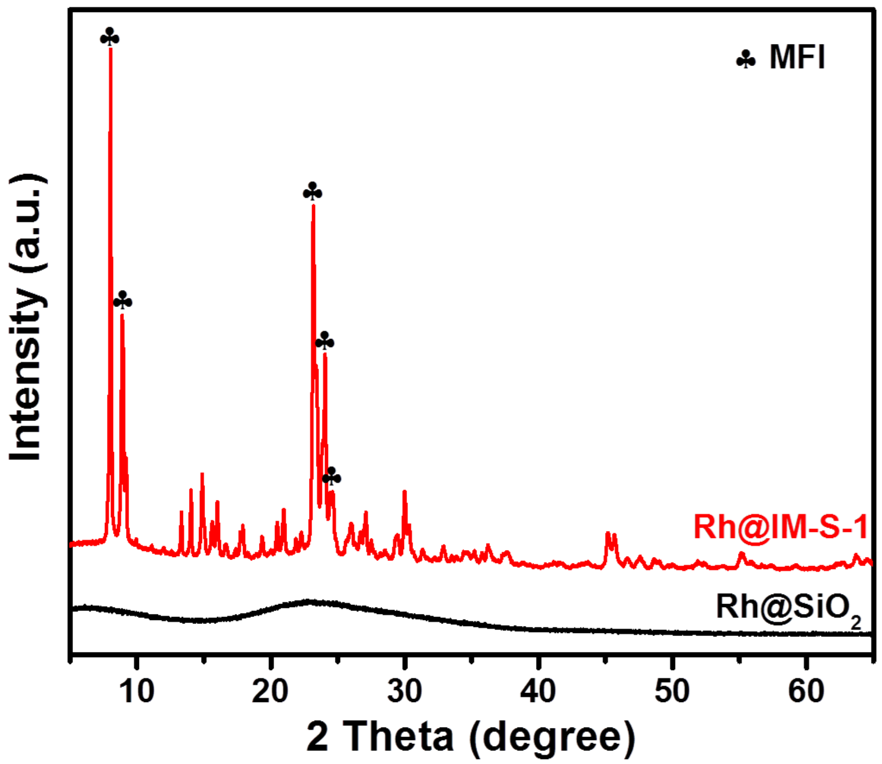

Supplementary Fig. 17 XRD patterns of 1%-Rh@SiO_2 and 1%-Rh@IM-S-1.

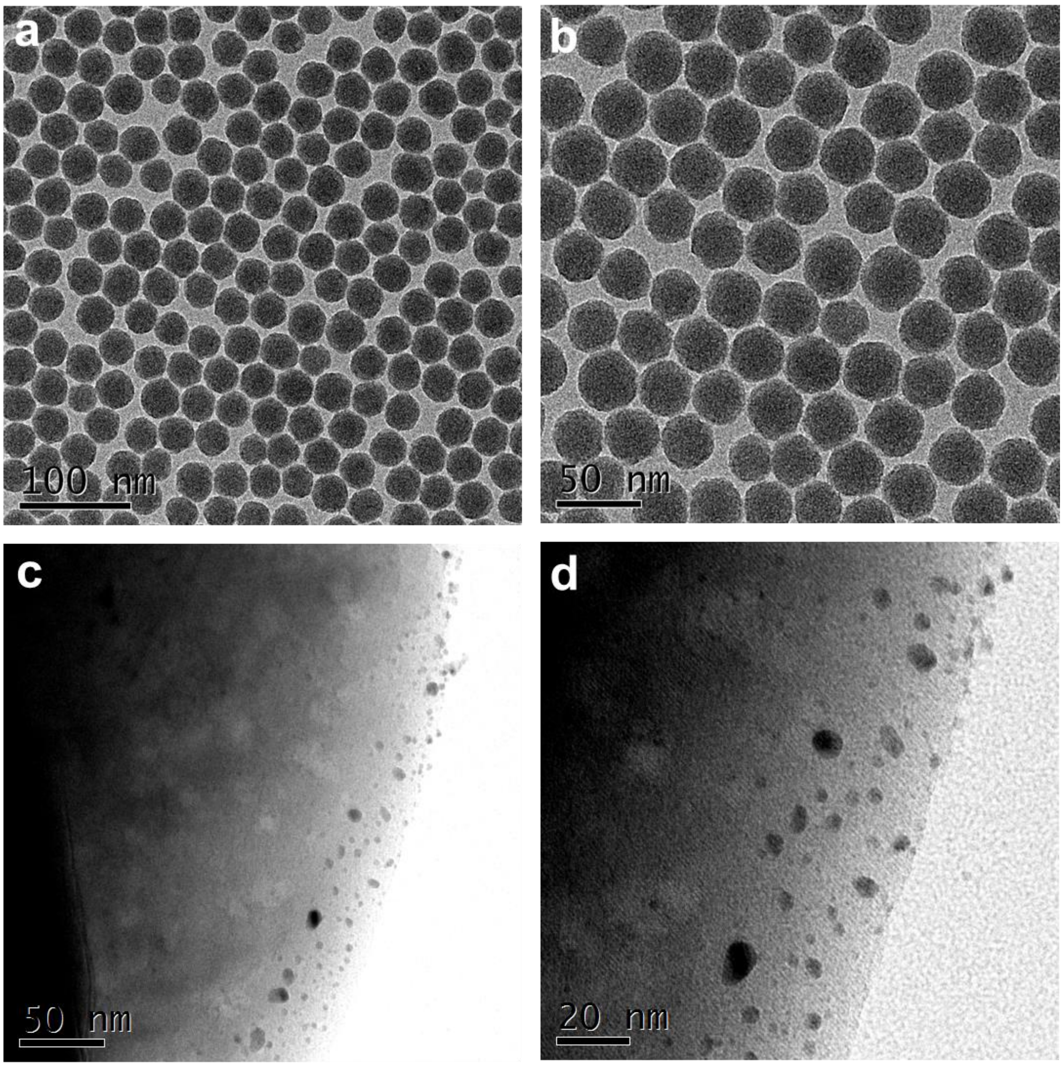

Supplementary Fig. 18 TEM of 1%-Rh@SiO_2 (a, b) and 1%-Rh@IM-S-1 (c, d).

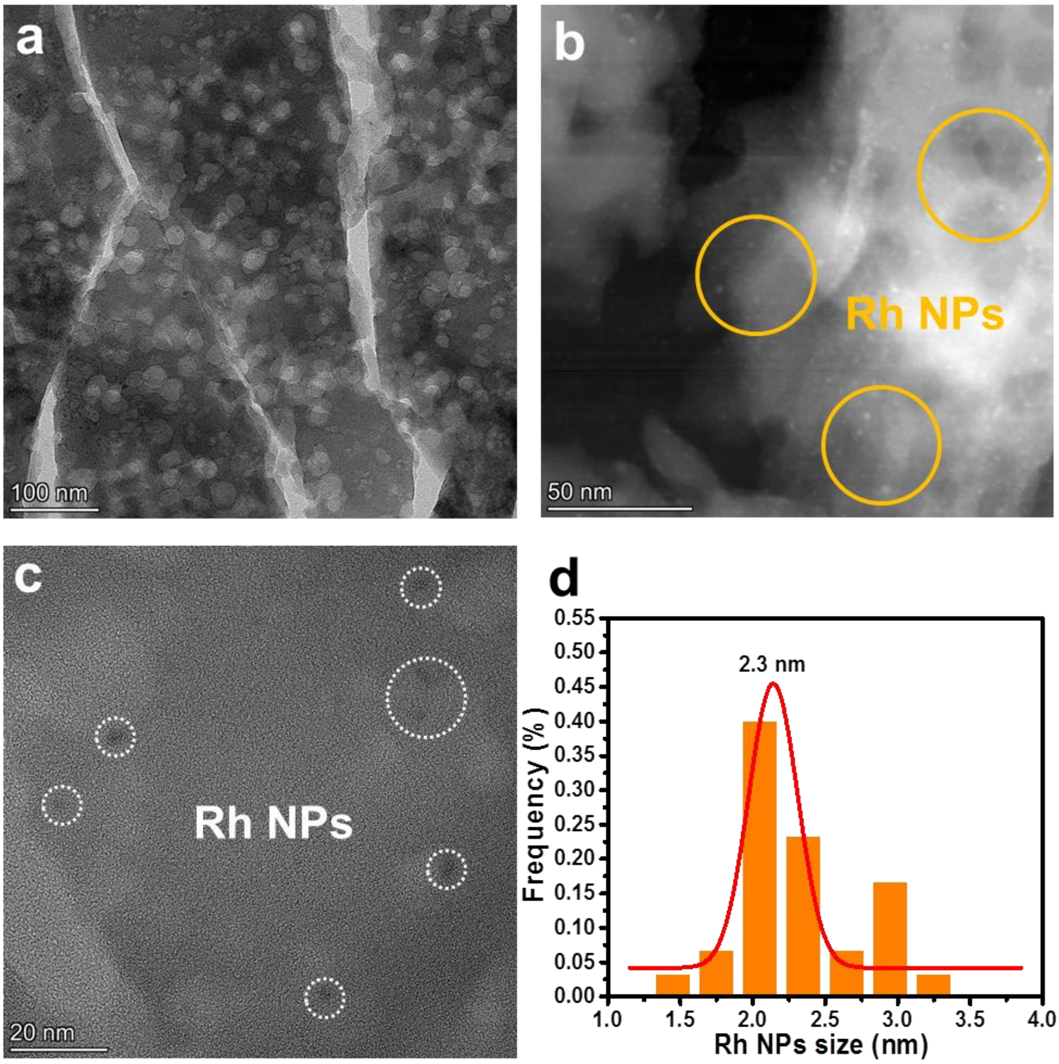

Supplementary Fig. 19 Tomogram-section TEM (a, c) and HAADF-STEM (b) images, and Rh NPs size distribution (d) of 1%-Rh@IM-S-1.

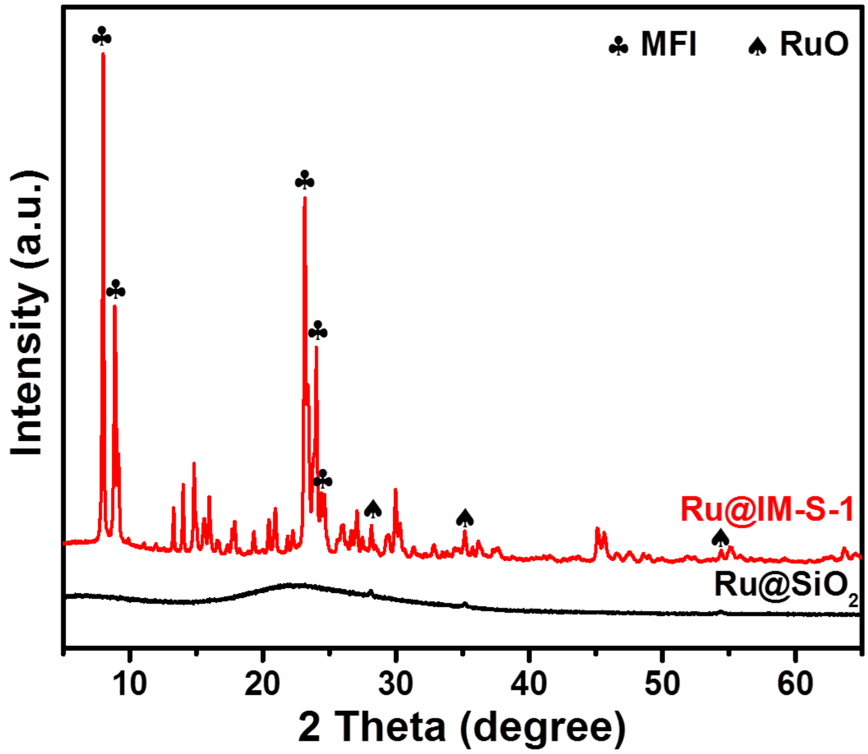

Supplementary Fig. 20 XRD patterns of 1%-Ru@SiO_2 and 1%-Ru@IM-S-1.

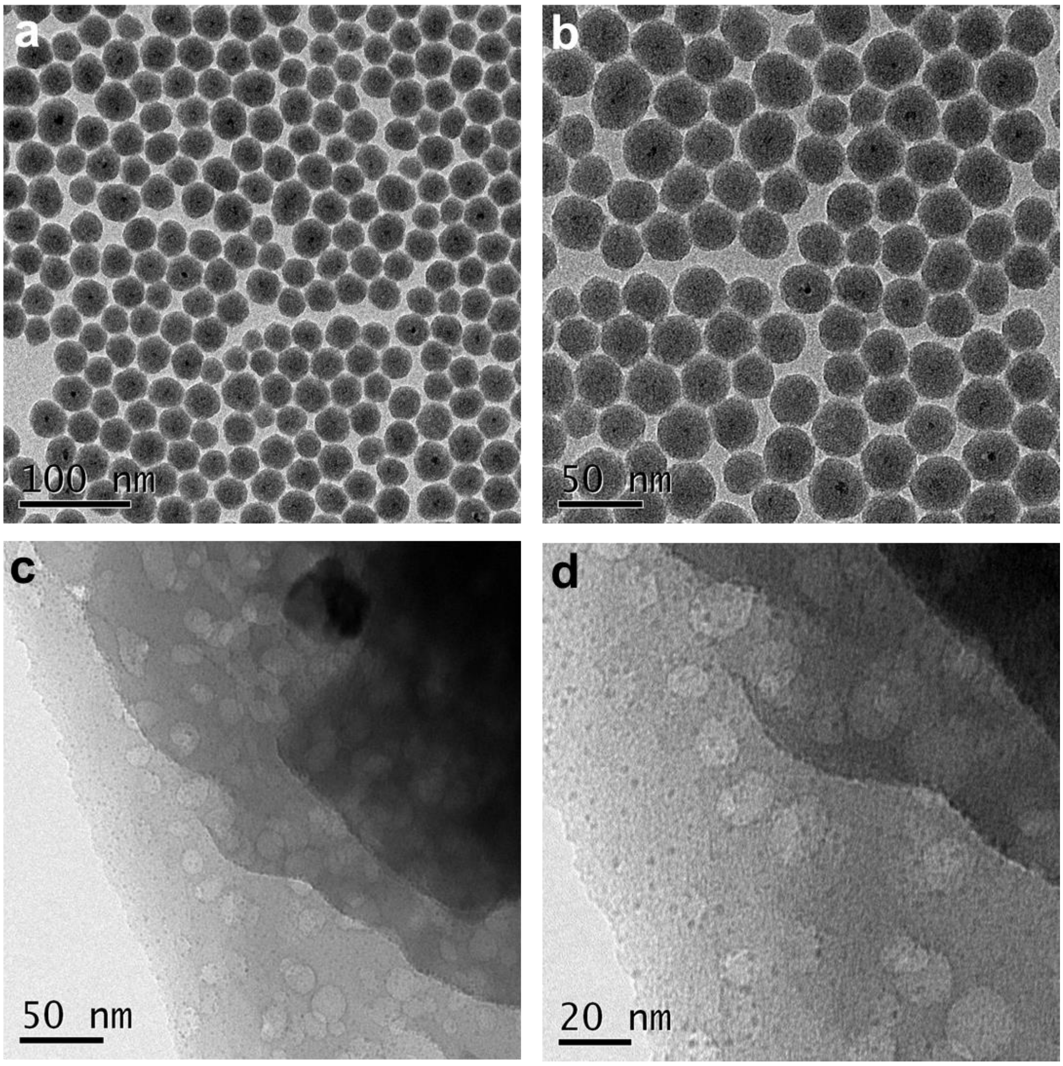

Supplementary Fig. 21 TEM of 1%-Ru@SiO_2 (a, b) and 1%-Ru@IM-S-1 (c, d).

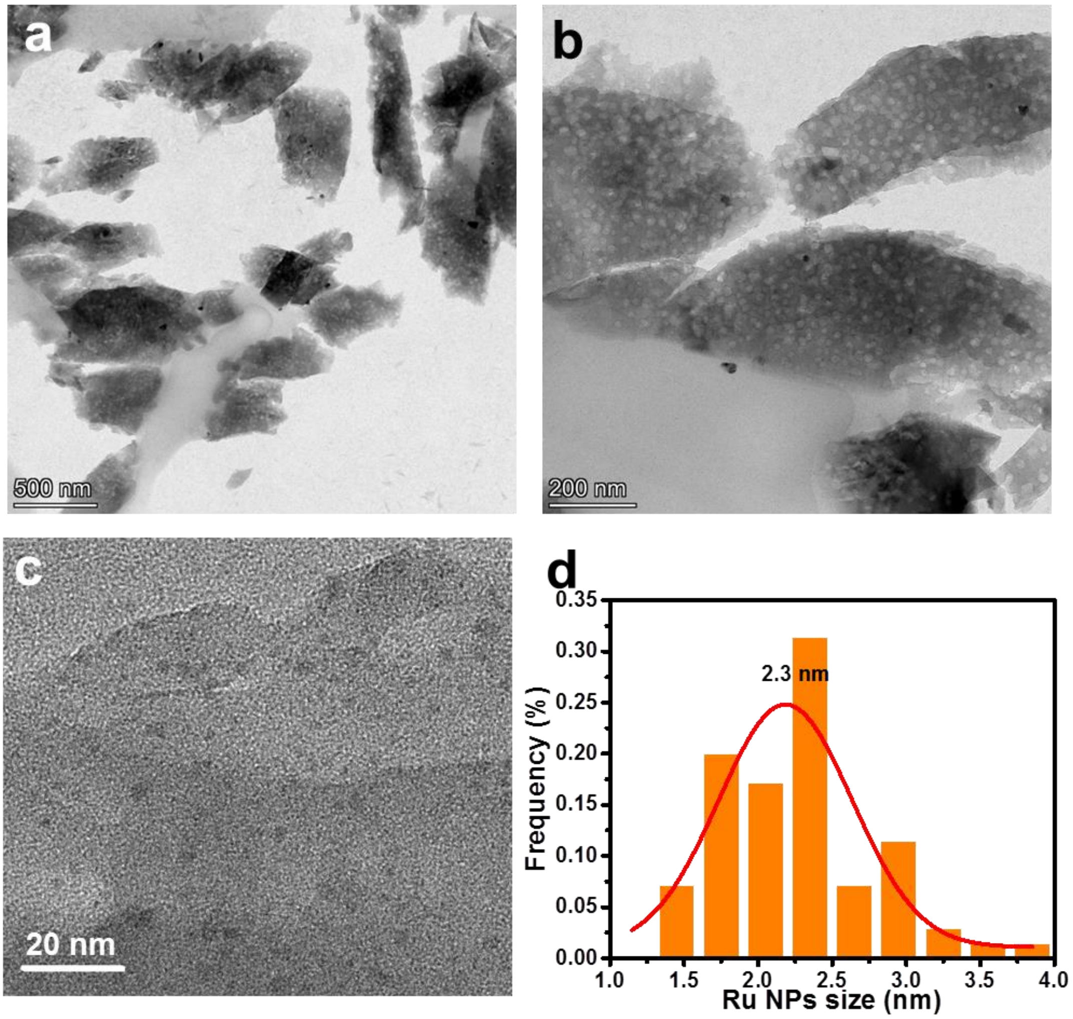

Supplementary Fig. 22 Tomogram-section TEM images (a, b, c) and Ru NPs size distribution (d) of 1%-Ru@IM-S-1.

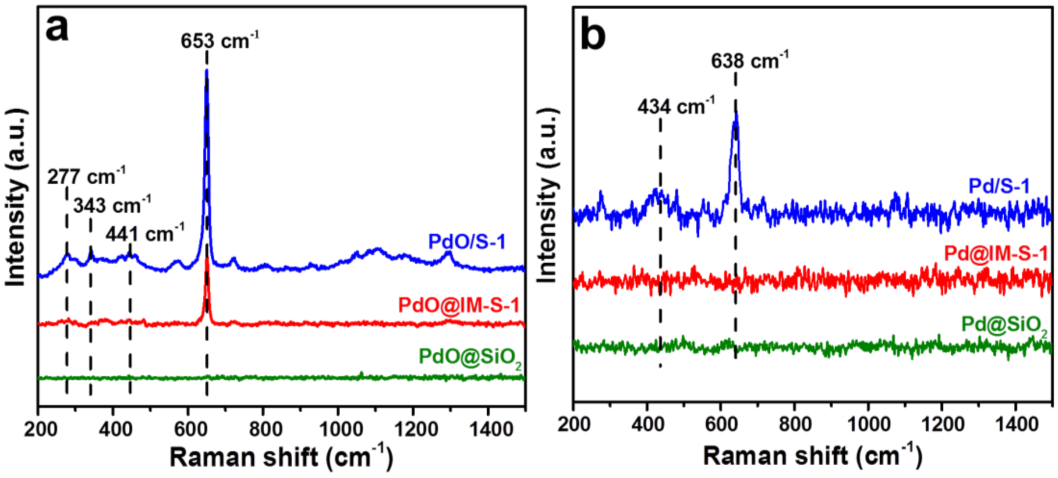

Supplementary Fig. 23 Raman spectra of unreduced samples PdO@IM-S-1, PdO/S-1, PdO@SiO_2 (a) and reduced samples Pd@IM-S-1, Pd/S-1, Pd@SiO_2 (b).

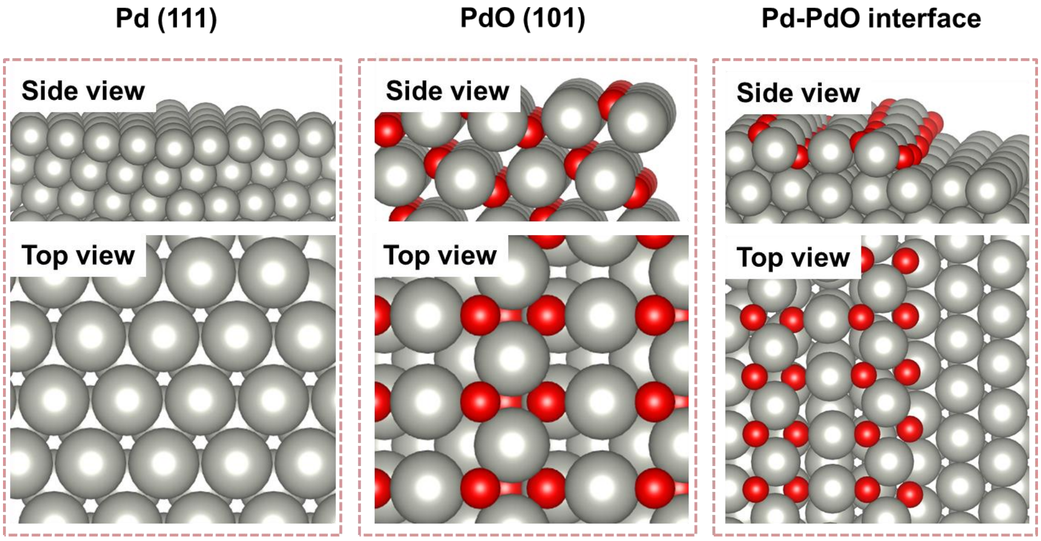

Supplementary Fig. 24 The optimized structure of Pd (111), PdO (101) and Pd-PdO interface.

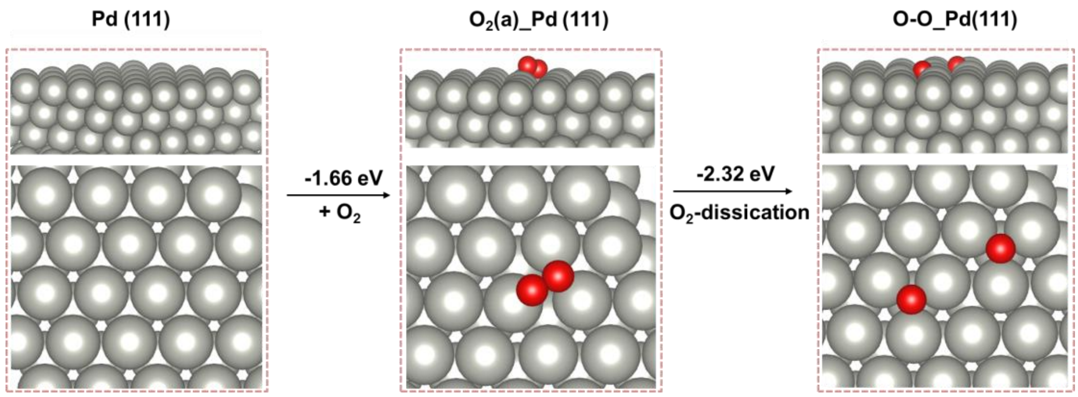

Supplementary Fig. 25 Oxygen dissociation process on Pd (111) slab.

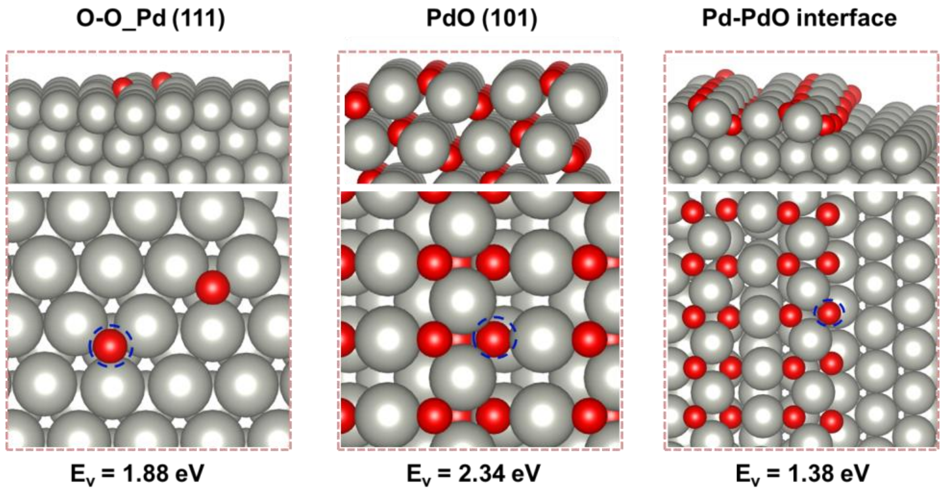

Supplementary Fig. 26 Oxygen vacancy formation energy (E_v) with different structure models.

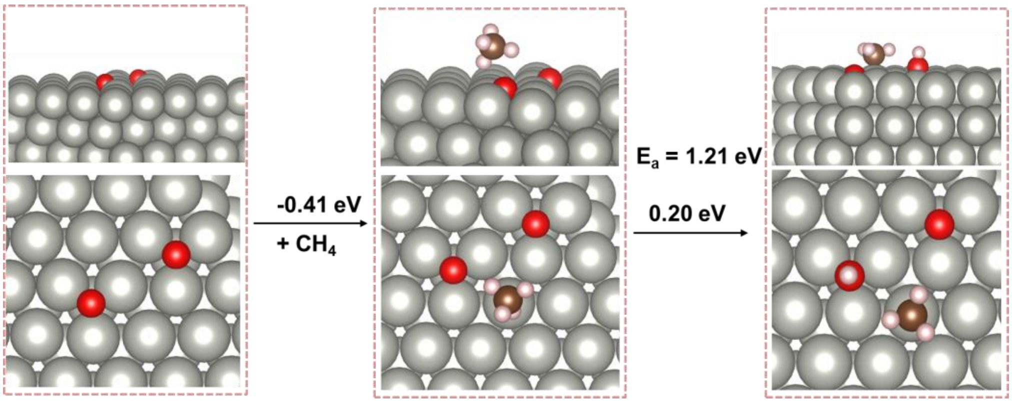

Supplementary Fig. 27 The activation energy (E_a) of the methane first C-H cleavage over the Pd (111) model.

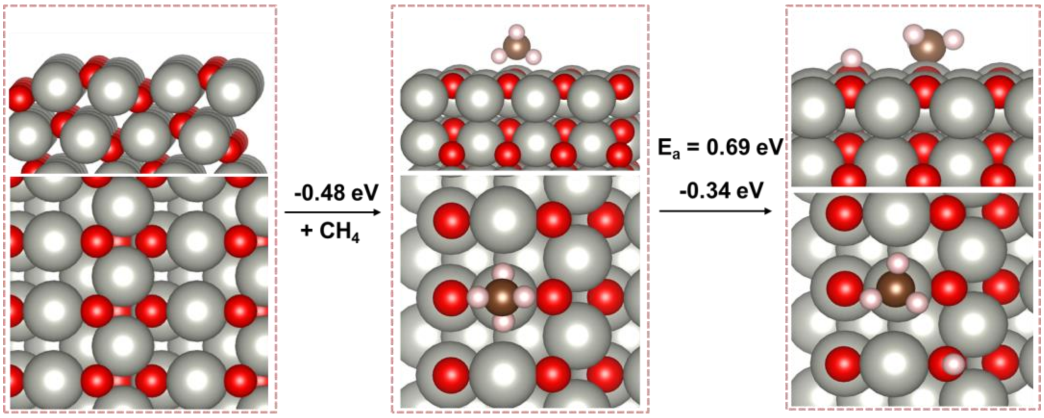

Supplementary Fig. 28 The activation energy (E_a) of the methane first C-H cleavage over the PdO (101) model.

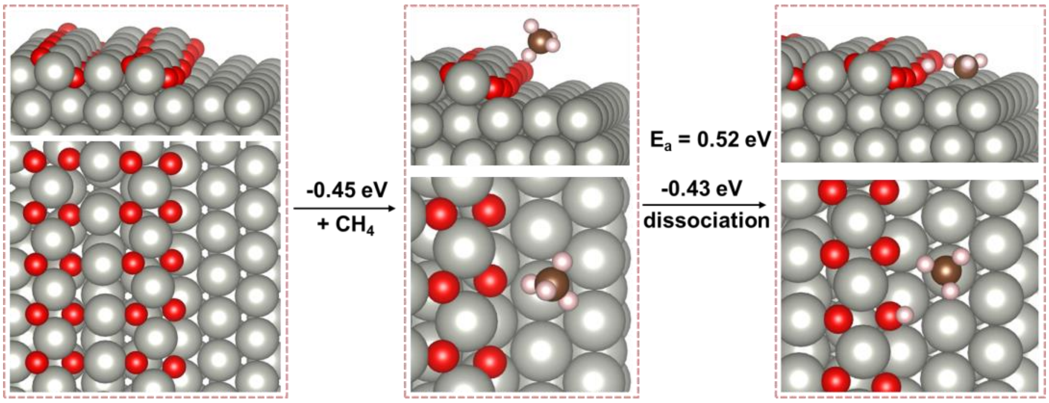

Supplementary Fig. 29 The activation energy (E_a) of the methane first C-H cleavage over the Pd-PdO interface model.

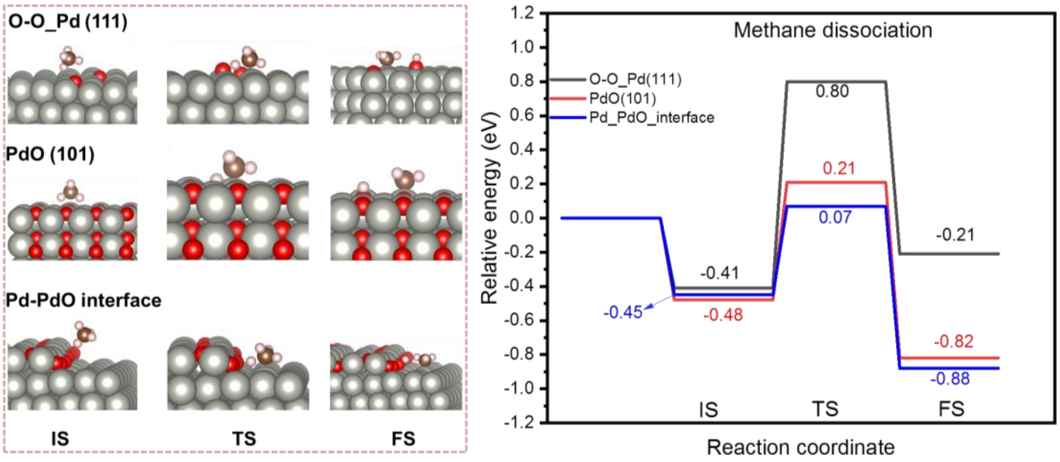

Supplementary Fig. 30 The possible reaction pathways of methane first C-H cleavage over these different structure models.

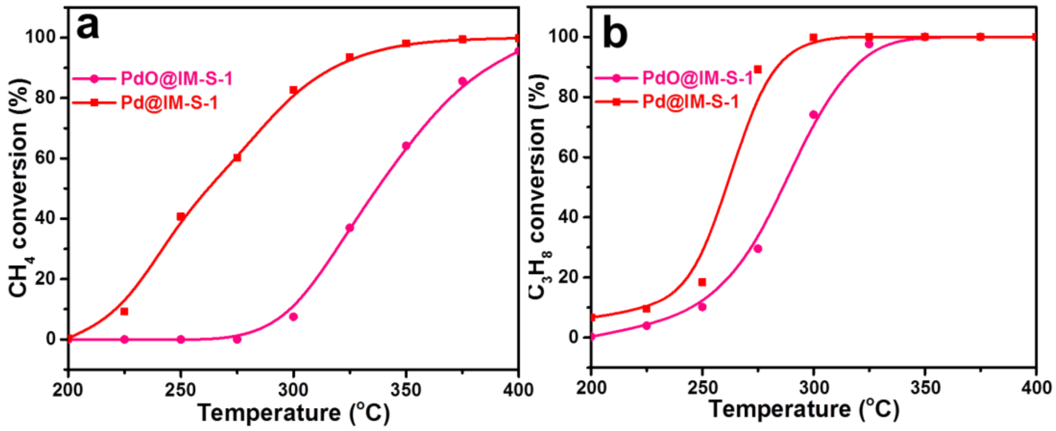

Supplementary Fig. 31 Deep oxidation performances of methane (a) and propane (b) over PdO@IM-S-1 and Pd@IM-S-1.

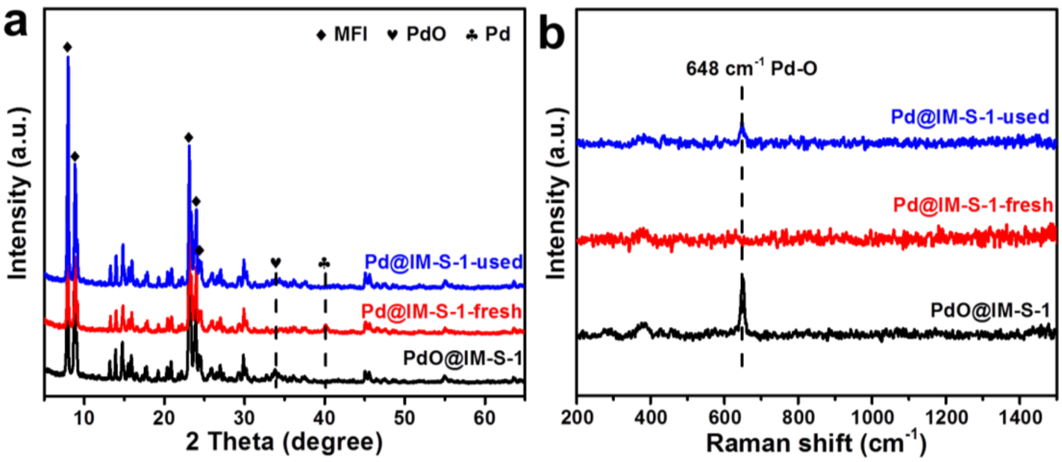

Supplementary Fig. 32 XRD patterns (a) and Raman spectra (b) of unreduced sample PdO@IM-S-1, unreacted sample Pd@IM-S-1-fresh, and reacted sample Pd@IM-S-1-used.

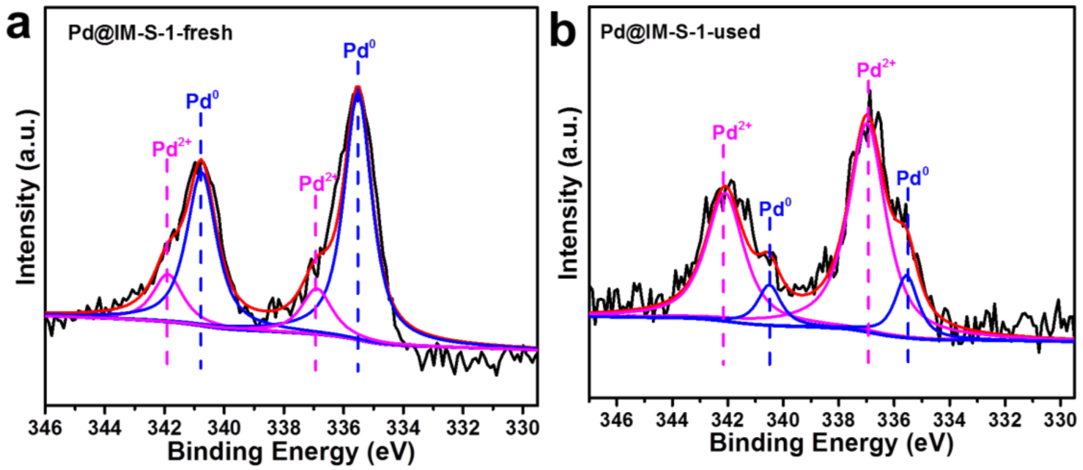

Supplementary Fig. 33 XPS of Pd3d for Pd@IM-S-1-fresh (a) and Pd@IM-S-1-used (b).

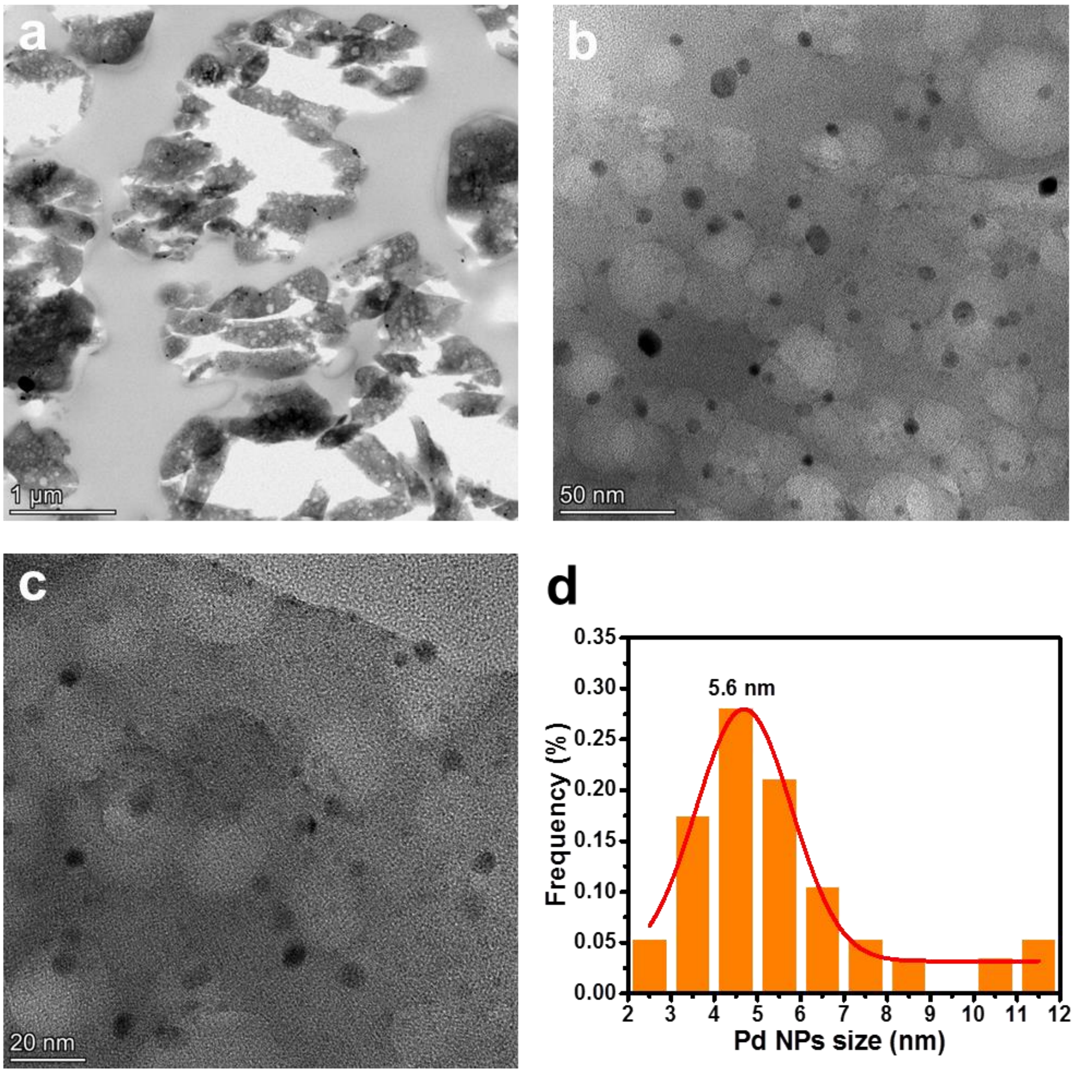

Supplementary Fig. 34 Tomogram-section TEM images (a, b, c) and Pd NPs size distribution (d) of Pd@IM-S-1-800.

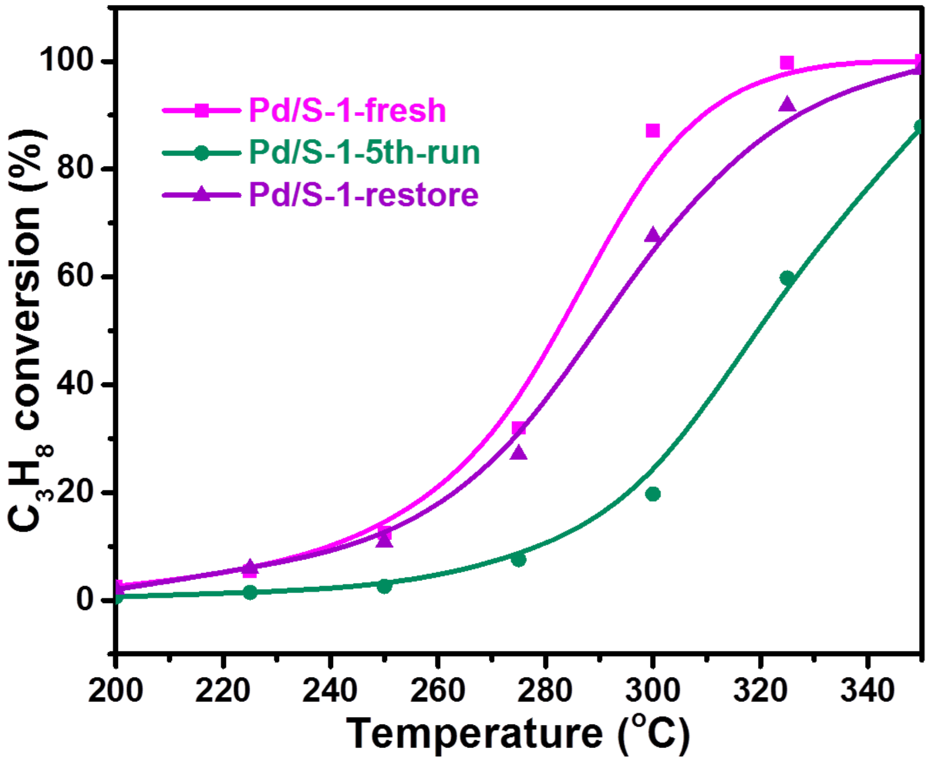

Supplementary Fig. 35 Regeneration test of Pd/S-1 in the oxidation of propane.

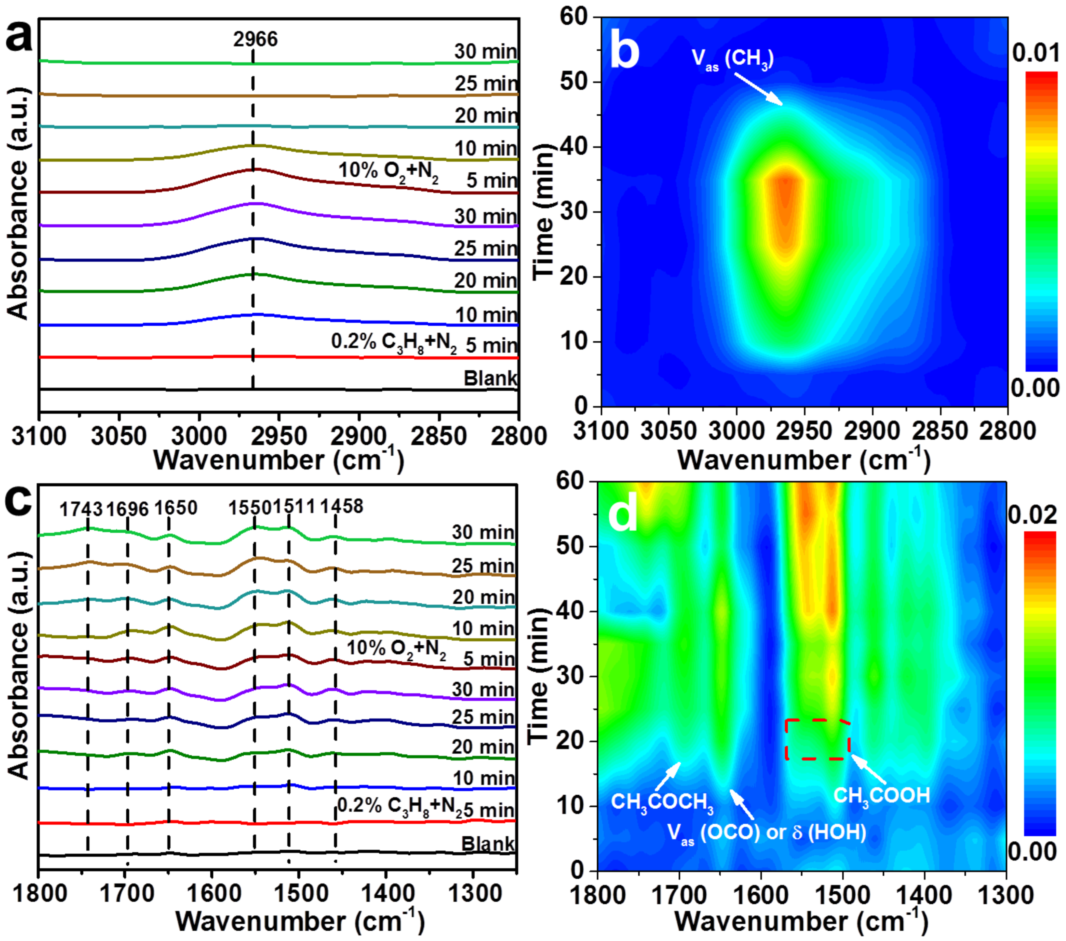

Supplementary Fig. 36 In situ DRIFTS spectra collected during propane deep oxidation over Pd@IM-S-1 at 325 °C (a, c). Images (b) and (d) are the corresponding mapping results of (a) and (c), respectively. Conditions: adsorption with 0.2% C_3H_8 + N_2 and then exposure in 10% O_2 + N_2 for 30 min; flow rate = 30 mL min^{-1}.

Supplementary Note 2: Based on the results presented in Supplementary Fig. 36a, b, the infrared absorption peak at 2966 cm^{-1} was attributed to the methyl asymmetric stretching $V_{as}(CH_3)$ of gaseous C_3H_8.[10, 19, 20] Supplementary Fig. 36c, d presents the spectra bands between 1300 and 1800 cm^{-1}; the assigned results are listed in Supplementary Table 10 in detail. The peaks located at 1743 and 1696 cm^{-1} can be attributed to the aliphatic ester and acetone.[11, 15] The peak located at 1650 cm^{-1} may be attributed to the $V_{as}(OCO)$ in bicarbonate (HCO_3) species or the δ(HOH) of molecularly adsorbed water.[11, 16, 17] Bands at 1550, 1511 cm^{-1} and 1458 cm^{-1} are likely due to the carboxylate group (COO) in bidentate acetate (CH_3COO) species.[10-14] The bands at 1550 and 1511 cm^{-1} are attributed to the asymmetric stretching of $V_{as}(COO)$, and the band at 1458 cm^{-1} is attributed to symmetric stretching of $V_s(COO)$.[10-14] In addition, the band intensities between 1300 and 1800 cm^{-1} (Supplementary Fig. 36c, d) show a gradual increase, while the bands between 2800 and 3000 cm^{-1} (Supplementary Fig. 36a, b) disappear when C_3H_8 gas is switched to 10% O_2. It was demonstrated that C_3H_8 oxidation occurred before the O_2 feed gas was introduced, demonstrating that the active oxygen species from the Pd–PdO interfaces participate in C_3H_8 deep oxidation consistent with the DFT calculation results and the Pd–PdO interfaces might be a new reaction site. Therefore, C_3H_8 deep oxidation over Pd@IM-S-1 might obey the Mars-van-Krevelen (MvK) mechanism.

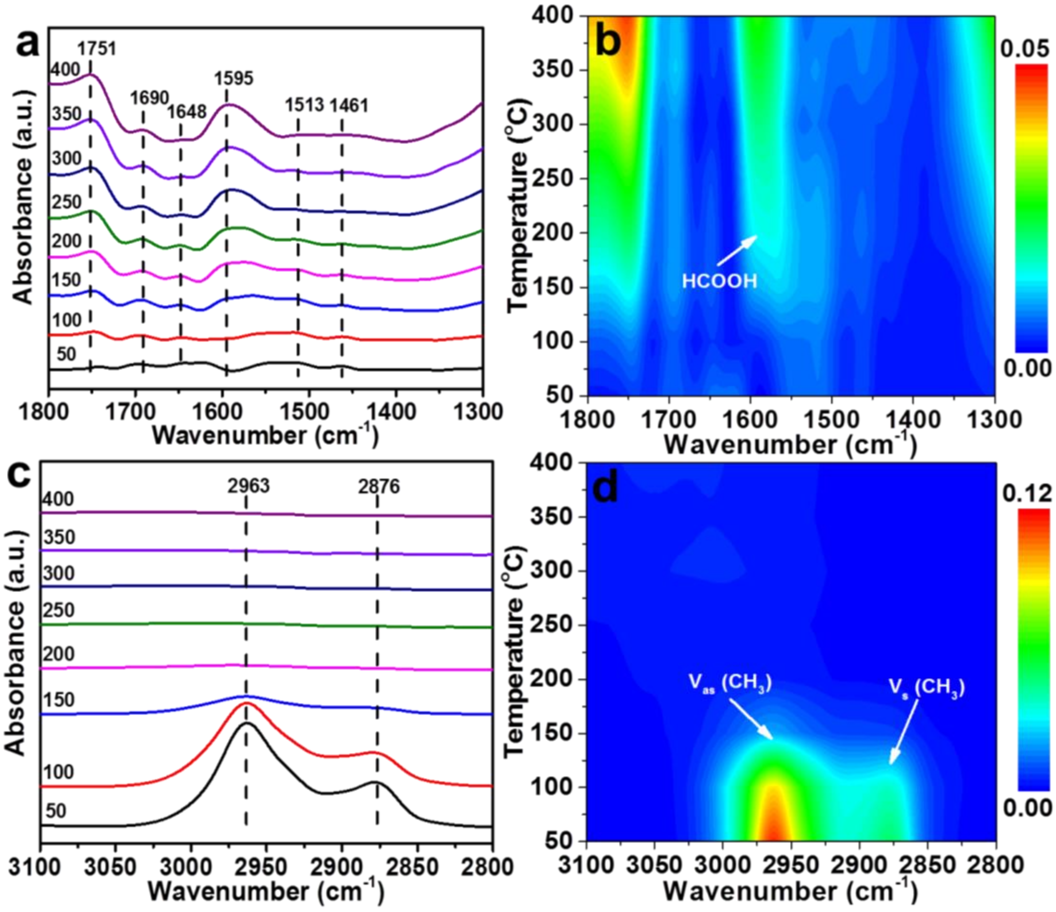

Supplementary Fig. 37 In situ DRIFTS spectra collected during propane temperature programed desorption (50 – 400 °C) in a N_2 + O_2 atmosphere over Pd@IM-S-1 (a, c). Images (b) and (d) are the corresponding mapping results of (a) and (c), respectively. Conditions: 2000 ppm C_3H_8 + N_2 for 30 min and then purging by highly pure N_2 for 30 min; 10% O_2 + N_2 was introduced at 50 °C, flow rate = 30 mL min^{-1}.

Supplementary Note 3: The C_3H_8-TPD testing over Pd@IM-S-1 in N_2 + O_2 flow gas was performed to investigate the conversion of the pre-adsorbed C_3H_8 species. The DRIFTS spectra were collected from 50 to 400 °C under a N_2 and O_2 mixture. The spectra bands in Supplementary Fig. 37a and b are attributed to the aliphatic ester at 1751 cm^{-1}, acetone at 1690 cm^{-1}, water or bicarbonate at 1648 cm^{-1}, formate of V_{as}(COO) at 1595 cm^{-1} [11, 18] and acetate at 1513 and 1461 cm^{-1}. The detailed assignments are listed in Supplementary Table 10. As shown in Supplementary Fig. 37c and d, the peaks at 2963 and 2876 cm^{-1} are associated with the gaseous C_3H_8 of $V_{as}(CH_3)$ and $V_s(CH_3)$. As the temperature increases, it can be seen that the bands at 2800–3000 cm^{-1} gradually weaken until they disappear. Meanwhile, the band intensities of the aliphatic ester, acetone, and formate increase slightly, suggesting that these species were difficult to desorb. However, the band intensity of the acetate species is slightly weakened, and the band intensity around water or bicarbonate gradually increases and then slightly decreases, revealing that these species were readily desorbed. These results indicate that the acetate and bicarbonate species might be considered as the active intermediates, and the aliphatic ester, acetone, and formate might be considered as the inert intermediates.

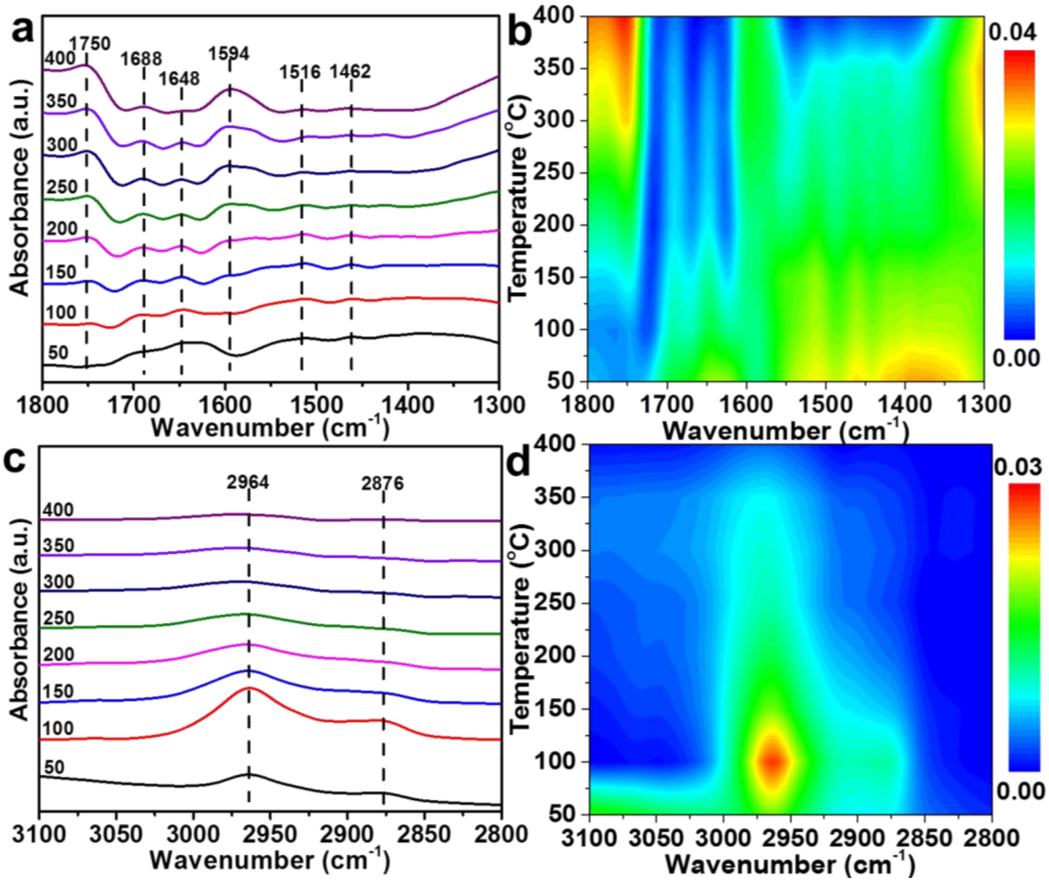

Supplementary Fig. 38 In situ DRIFTS spectra collected from the C_3H_8 + O_2 reaction over Pd@IM-S-1 between 50 °C and 400 °C (a, c). Images (b) and (d) are the corresponding mapping results of (a) and (c), respectively. Reaction conditions: 2000 ppm C_3H_8 + 10% O_2 + N_2, flow rate = 30 mL min^{-1}.

Supplementary Note 4: To further study the C_3H_8 deep oxidation process over Pd@IM-S-1, C_3H_8-TPO was tested, and the DRIFTS spectra were collected at 50–400 °C via propane and O_2 co-adsorption. As presented in Supplementary Fig. 38a and b, the band intensity around bicarbonate (1647 cm^{-1}) gradually increases and then decreases. But the band intensity around the aliphatic ester (1750 cm^{-1}), acetone (1688 cm^{-1}), and formate (1597 cm^{-1}) always increases; and that of the acetate (1516 cm^{-1}, and 1462 cm^{-1}) gradually decreases. Significantly, as the temperature increases, the band intensity around gaseous C_3H_8 (2800-3000 cm^{-1}) gradually increases and then decreases, as shown in Supplementary Fig. 38c and d. These results demonstrate that Pd@IM-S-1 has the trapping and concentrating ability of C_3H_8 at low temperatures and activates it at high temperatures. These results are similar to those shown in Supplementary Fig. 37a and b; and they further confirm that acetate and bicarbonate species should be considered as the active intermediates and the aliphatic ester, acetone, and formate should be considered as the inert intermediates.

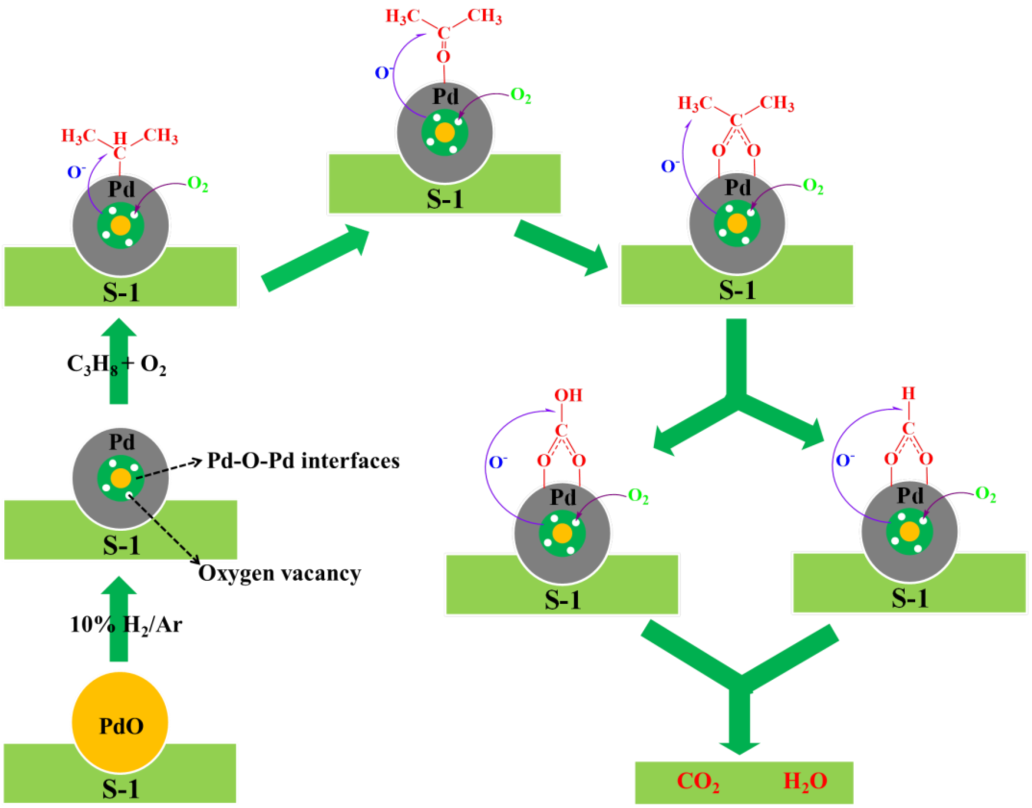

Supplementary Fig. 39 Possible reaction mechanism for the oxidation of propane on Pd@IM-S-1 catalyst.

Supplementary References

1. Peng, H. et al. Active and stable Pt-Ceria nanowires@silica shell catalyst: Design, formation mechanism and total oxidation of CO and toluene. *Appl. Catal. B-Environ.* **256**, 117807 (2019).
2. Peng, H. et al. Confined ultrathin Pd-Ce nanowires with outstanding moisture and SO_2 tolerance in methane combustion. *Angew. Chem. Int. Ed.* **57**, 8953-8957 (2018).
3. Zhang, Q. et al. High-quality single-crystalline MFI-type nanozeolites: a facile synthetic strategy and MTP catalytic studies. *Chem. Mater.* **30**, 2750-2758 (2018).
4. Ma, R., Wang, L., Wang, S., Wang, C. & Xiao, F. Eco-friendly photocatalysts achieved by zeolite fixing. *Appl. Catal. B-Environ.* **212**, 193-200 (2017).
5. Lippert, G., Hutter, J. & Parrinello, M. The gaussian and augmented-plane-wave density functional method for ab initio molecular dynamics simulations. *Theor. Chem. Acc.* **103**, 124-140 (1999).
6. VandeVondele, J., Krack, M., Mohamed, F., Parrinello, M., Chassaing, T. & Hutter, J. Quickstep: fast and accurate density functional calculations using a mixed gaussian and plane waves approach. *Comput. Phys. Commun.* **167**, 103-128 (2005).
7. Burke, K., Ernzerhof, M. & Perdew, J. P. Generalized gradient approximation made simple. Phys. Rev. Lett. 77, 3865 (1996).
8. Teter, M., Hutter, J. & Goedecker, S. Separable dual-space gaussian pseudopotentials. *Phys. Rev. B* **54**, 1703-1710 (1996).
9. Grimme, S., Antony, J., Ehrlich, S. & Krieg, H. A consistent and accurate ab initio parametrization of density functional dispersion correction (DFT-D) for the 94 elements H-Pu. *J. Chem. Phys*. **132**, 154104 (2010).
10. Hu, Z. et al. Total oxidation of propane over a Ru/CeO_2 catalyst at low temperature. *Environ. Sci. Technol.*, **52**, 9531-9541 (2018).
11. O'Brien, C. P. & Lee, I. C. A detailed spectroscopic analysis of the growth of oxy-carbon species on the surface of Pt/Al_2O_3 during propane oxidation. *J. Catal.* **347**, 1-8 (2017).
12. Yu, Y., He, H., Zhang, X. & Deng, H. A common feature of H_2-assisted HC-SCR over Ag/Al_2O_3. *Catal. Sci. Technol.* **4**, 1239-1245 (2014).
13. Haneda, M. et al. In Situ Fourier Transform Infrared study of the selective reduction of NO with propene over Ga_2O_3-Al_2O_3. *J. Catal.* **206**, 114-124 (2002).
14. Captain, D. K. & Amiridis, M. D. In Situ FTIR Studies of the selective catalytic reduction of NO by C_3H_6 over Pt/Al_2O_3. *J. Catal.* **184**, 377-389 (1999).
15. Airaksinen, S. M. K., Bañares, M. A. & Krause, A. O. I. In situ characterisation of carbon-containing species formed on chromia/alumina during propane dehydrogenation. *J. Catal.* **230**, 507-513 (2005).
16. Guerrero, S., Miller, J. T., Kropf, A. J. & Wolf, E. E. In situ EXAFS and FTIR studies of the promotion behavior of Pt-Nb_2O_5/Al_2O_3 catalysts during the preferential oxidation of CO. *J. Catal.* **262**, 102-110 (2009).
17. Schubert, M. M., Gasteiger, H. A. & Jürgen Behm, R. Surface formates as side products in the selective CO oxidation on Pt/γ-Al_2O_3. *J. Catal.* **172**, 256-258 (1997).
18. Matsouka, V., Matsouka, V., Konsolakis, M., Lambert, R. M. & Yentekakis, I. V. In situ DRIFTS study of the effect of structure (CeO_2-La_2O_3) and surface (Na) modifiers on the catalytic and surface behaviour of Pt/γ-Al_2O_3 catalyst under simulated exhaust conditions. *Appl. Catal. B-Environ.* **84**, 715-722 (2008).
19. Wang, B., Wu, X., Ran, R., Si, Z. & Weng, D. IR characterization of propane oxidation on Pt/CeO_2-ZrO_2: The reaction mechanism and the role of Pt. *J. Mol. Catal. A-Chem*. **356**, 100-105 (2012).
20. Hoost, T. E., Laframboise, K. A. & Otto, K. Co-adsorption of propene and nitrogen oxides on Cu-ZSM-5: an FTIR study. *Appl. Catal. B-Environ.* **7**, 79-93 (1995).
